# Biochemical and Genomic Underpinnings of Carotenoid Colour Variation Across a Hybrid Zone Between South Asian Flameback Woodpeckers

**DOI:** 10.1111/mec.70084

**Published:** 2025-08-19

**Authors:** Rashika W. Ranasinghe, Jocelyn Hudon, Sampath S. Seneviratne, Darren Irwin

**Affiliations:** ^1^ Department of Zoology and Biodiversity Research Centre University of British Columbia Vancouver British Columbia Canada; ^2^ Royal Alberta Museum Edmonton Alberta Canada; ^3^ Avian Sciences & Conservation, Department of Zoology & Environment Sciences, Faculty of Science University of Colombo Colombo Sri Lanka

**Keywords:** carotenoids, *CYP2J2*, *Dinopium*, GWAS, hybrids, Sri Lanka

## Abstract

Colouration and patterning have been implicated in lineage diversification across various taxa, as colour traits are heavily influenced by sexual and natural selection. Investigating the biochemical and genomic foundations of these traits therefore provides deeper insights into the interplay between genetics, ecology and social interactions in shaping the diversity of life. In this study, we assessed the pigment chemistries and genomic underpinnings of carotenoid colour variation in naturally hybridising *Dinopium* flamebacks in tropical South Asia. We employed reflectance spectrometric analysis to quantify species‐specific plumage colouration, High‐Performance Liquid Chromatography (HPLC) to elucidate the feather carotenoids of flamebacks across the hybrid zone, and Genome‐Wide Association Study (GWAS) using next‐generation sequencing data to uncover the genetic factors underlying carotenoid colour variation in flamebacks. Our analysis revealed that the red mantle feathers of *D. psarodes* primarily contained astaxanthin, with small amounts of other 4‐keto‐carotenoids. In contrast, the yellow mantle feathers of *D. benghalense* predominantly contained lutein and 3′‐dehydro‐lutein, alongside minor amounts of zeaxanthin, β‐cryptoxanthin and canary‐xanthophylls A and B. Hybrids with an intermediate, orange colouration deposited all of these pigments in their mantle feathers, with notably higher concentrations of carotenoids with ε‐end rings. The GWAS analysis identified the *CYP2J2* gene, which plays a role in carotenoid ketolation, as associated with the expression of carotenoid colouration. Read depth data suggested variation in copy number of this gene in flamebacks. These findings contribute to the growing knowledge of avian carotenoid metabolism and highlight how genomic architecture can influence phenotypic diversity.

## 1 Introduction

Colouration and patterning in organisms are instrumental to lineage diversification as these traits are strongly shaped by sexual and natural selection (Cuthill et al. 2017; Delhey et al. 2023; Hill and McGraw 2006; Negro et al. 1998; Orteu and Jiggins 2020; Postema et al. 2023; Svensson and Wong 2011; Willink and Wu 2023). Investigating colouration and patterns, therefore, provides valuable insights into the mechanisms of inter‐ and intra‐species interactions, adaptive evolution and speciation (Caro and Allen 2017; Chamberlain et al. 2009; Eliason et al. 2023, 2024; Hubbard et al. 2010; Orteu and Jiggins 2020; Price‐Waldman and Stoddard 2021; Roulin 2007). Avian taxa are particularly suited for exploring these phenomena because of the rapid evolution of plumage colouration, as evidenced by closely related species exhibiting conspicuous and distinct colouration patterns (Campagna et al. 2012; Milá et al. 2007; Ödeen and Björklund 2003; Semenov et al. 2018).

Striking avian colourations are generated through the deposition of a variety of pigments, such as melanins, carotenoids, porphyrins, pterins and psittacofulvins, or through structural specialisations of feathers or combinations of both (Cooke et al. 2017; Hill and McGraw 2006; Justyn and Weaver 2023; Mundy 2018; Price‐Waldman and Stoddard 2021; Shawkey and D'Alba 2017; Shawkey and Hill 2005; Toews et al. 2017). The vibrant yellow, orange and red hues in bird plumages are typically produced by carotenoid pigments (Hill and McGraw 2006). Carotenoids are hydrocarbon molecules characterised by a backbone of conjugated double bonds capped by two terminal end rings bearing various functional groups (Britton 1995; Fernandes 2018; Goodwin 1986). The colour of these molecules is largely a function of the length of the conjugated double‐bond system determining their light absorption properties. Functional groups of the end rings can either elongate or shorten this conjugated system, thereby shifting their peak of absorption. Molecules with longer conjugated double‐bond systems exhibit peak absorbance at longer wavelengths, often producing redder hues, while molecules with shorter such systems have peak absorbances at shorter wavelengths, resulting in yellower hues. Animals cannot endogenously synthesise carotenoids, and they must acquire them through their diet (Maoka 2020; McGraw 2006). Once ingested, dietary carotenoids—such as lutein, zeaxanthin, β‐cryptoxanthin and β‐carotene—can be metabolically modified to produce diverse pigments with new spectral properties (Brush 1990; McGraw 2006; Stradi 1998). Beyond their role in visual signalling, carotenoids may also confer antioxidant benefits, potentially contributing to immune defence mechanisms and other physiological functions (Alonso‐Alvarez et al. 2004; Lozano 1994; McLean et al. 2005), although there is debate regarding the strength of evidence for these roles in birds (Koch and Hill 2018; Koch et al. 2018). β‐carotene also serves as a precursor of vitamin A, which is essential for growth, development and vision in animals (Hill and Johnson 2012). The possible tie of carotenoid metabolism to core physiological functions like cellular respiration (Cantarero et al. 2020; Fernández‐Eslava et al. 2023; Hill 2011, 2014; Hill and Johnson 2012; Johnson and Hill 2013; Weaver et al. 2018) and potential trade‐offs between colouration, immune and other functions have made carotenoid expression a prime subject of research into honest signalling of individual quality and health (Alonso‐Alvarez et al. 2004, 2009; McGraw et al. 2005; Svensson and Wong 2011).

The characterisation of genes involved in carotenoid expression and metabolism has been challenging due to the diversity of pigments and functions across taxa, the complexity of biochemical processing, and the influence of environmental factors on carotenoid biochemistry in organisms (Matrková and Remeš 2012; Muller et al. 2012; Toews et al. 2017). However, recent advances in molecular and genomic technologies have enabled the identification of genes involved in carotenoid expression in different organisms (Funk and Taylor 2019; Hooper et al. 2024; Lim et al. 2024; Toews et al. 2017). Key genes discovered include those involved in carotenoid uptake: *SCARB1*‐*2,15* (Kiefer et al. 2002; Toomey et al. 2017), *SCARF2* (Brelsford et al. 2017); binding and deposition: *StAR1*, *MLN64*, *StAR4*, *StAR5*, *APOD*, *PLIN*, *GSTA2* (Walsh et al. 2012); modification: *CYP2J19* (Lopes et al. 2016; Mundy et al. 2016), *BDH1L*, *TTC39B* (Hooper et al. 2024; Toomey et al. 2022); and degradation: *BCO1‐2* (Eriksson et al. 2008; Gazda et al. 2020; Pointer and Mundy 2008; Walsh et al. 2012). Understanding the processes and reactions mediated by these genes is crucial to unravelling how carotenoid pigments are absorbed, modified and utilised within the body, as well as their ecological and evolutionary significance (Roulin and Ducrest 2013). The identification and quantification of carotenoid pigments are essential to comprehending signal content and evolution, as well as understanding the ecological and social selection pressures on carotenoid displays.

**FIGURE 1:**
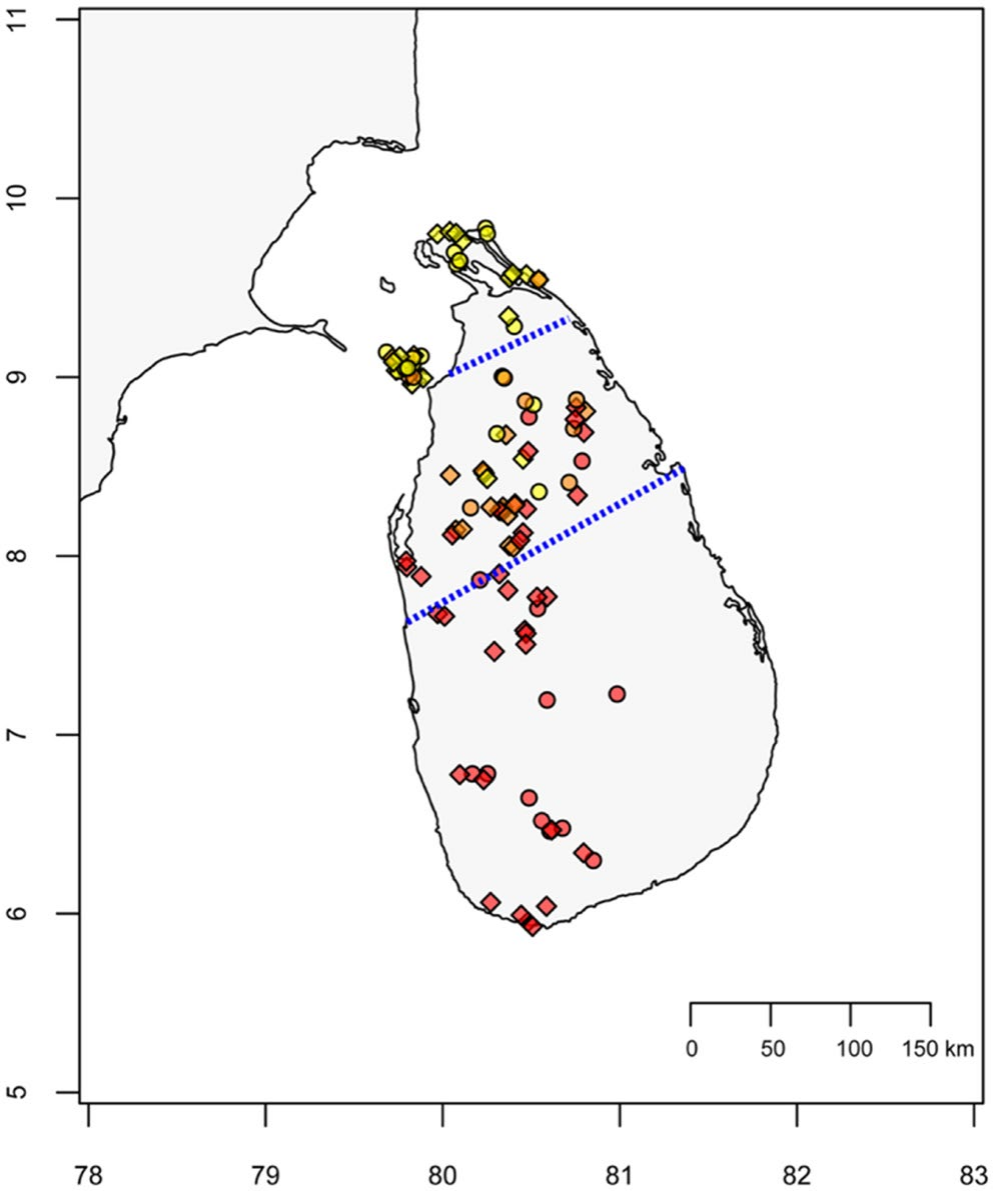
*Dinopium* flameback hybrid zone in Sri Lanka. Blue dashed lines indicate the boundaries of the contact zone between the two species— *D. psarodes* in the south and *D. benghalense* in the north. Coloured points represent individual samples for GWAS analysis (*n* = 106) with red, yellow and orange indicating red‐backed *D. psarodes* , yellow‐backed *D. benghalense* and orange‐backed intermediate flamebacks, respectively. Circular markers denote individuals from which mantle feathers were sampled for carotenoid pigment analysis (*n* = 40—a subset of GWAS samples), while diamonds represent other GWAS samples. Crown feathers used for carotenoid analysis were taken from a subset of individuals sampled for mantle feathers. Sample coordinates have been slightly jittered to reduce overlap.

The conspicuous, naturally hybridising *Dinopium* flameback woodpeckers of Sri Lanka provide an excellent system to investigate the biochemical and genetic mechanisms underlying carotenoid colour expression. The island of Sri Lanka hosts two species of *Dinopium* flamebacks: the endemic *D. psarodes* (Red‐backed Flameback), characterised by striking crimson‐red colouration on the mantle, coverts, secondaries and head; and *D. benghalense* (Black‐rumped Flameback), which displays golden‐yellow colouration on the mantle, coverts, secondaries along with a crimson‐red head (delHoyo et al. 2002; Fernando et al. 2016; Fernando and Seneviratne 2015). The two species hybridise extensively in areas of sympatry (Figure 1), resulting in individuals that exhibit a continuum of intermediate orange hues on the mantle, coverts and secondaries, while retaining the crimson‐red head (Fernando and Seneviratne 2015; Freed et al. 2015). The two species, however, show little genetic differentiation between them (Ranasinghe et al. 2024). This natural gradient of plumage colouration from red to orange to yellow, combined with extensive genomic admixture and shallow genetic differentiation (Ranasinghe et al. 2024), offers an ideal system to examine the genetic basis of carotenoid colour expression. Furthermore, the recent discovery of cryptic divergence within the yellow‐backed *D. benghalense* species (Ranasinghe et al. 2024) actually suggests three‐way hybridisation among *Dinopium* flamebacks on the island. This discovery further enhances the system's potential for dissecting the genetic architecture of red versus yellow carotenoid colouration. In the present study, we characterise the carotenoid composition of red, yellow and various shades of orange mantle feathers of flamebacks across the hybrid zone. Based on the pigment profiles, we propose probable carotenoid metabolic pathways in *Dinopium* species. Furthermore, we integrate genomic data and admixture mapping to identify genetic variants that are associated with divergent carotenoid‐based colour expression in birds.

## 2 Methods

### 2.1 Sample Collection

We captured red‐backed *D. psarodes*, yellow‐backed *D. benghalense* and orange‐backed intermediate flameback woodpeckers across the *Dinopium* hybrid zone in Sri Lanka (Fernando et al. 2016; Fernando and Seneviratne 2015; Ranasinghe et al. 2024). The birds were captured using mist nets, with the aid of lifelike wooden‐crafted flameback decoys and playbacks of locally recorded flameback vocalisations to attract individuals into the nets. From each captured bird, we collected approximately 50 μL of blood from the brachial vein into 500 μL of Queen's lysis buffer (0.01 M Tris, 0.01 M NaCl, 0.01 M EDTA, 1% n‐lauroyl sarcosine, pH 7.5) for subsequent DNA extraction and sequencing for GWAS analysis. We also collected three crown feathers and three mantle feathers from each bird. The feather samples were placed in brown envelopes and stored in the refrigerator until pigment extraction. In total, we obtained mantle feathers from 40 flamebacks (13 red‐backed *D. psarodes* , 19 yellow‐backed *D. benghalense* , and eight orange‐backed intermediate‐coloured flamebacks) and red crown feathers from 20 birds (8 *D. psarodes* , 9 *D. benghalense* and 3 intermediate‐coloured flamebacks; Figure 1).

### 2.2 Feather Reflectance Spectroscopy and Colour Metrics

We performed reflectance spectrometry using an Ocean Optics USB2000+ spectrophotometer equipped with a bifurcated optic fibre reflection probe (R400‐7‐UV‐VIS, Ocean Optics) and a Mikropack DH–2000‐BAL deuterium/tungsten/halogen light source. The probe was positioned 1.9 mm directly above the feather surface at a 90° angle, utilising a non‐reflective probe holder (RPH‐1) for alignment. Prior to measurement, we calibrated the spectrometer with a 99% Spectralon white standard (WS‐1‐SL, Ocean Optics), turning the light source off as our dark standard. With each sample, we stacked three feathers on top of each other on a black velvet background and measured the reflectance. We acquired three replicate reflectance spectra from each sample, and we analysed and visualised the data within the 375–700 nm range using the *pavo* package in R‐version 4.4.0 (Maia et al. 2019; R Core Team 2024).

We calculated three main colour metrics: (1) Hue, as *λ*_R50_, the wavelength at which reflectance (*R*) is halfway between the minimum at mid‐wavelengths and the maximum at longer wavelengths between 450 and 700 nm (also known as *R*_mid_); (2) Mean brightness, mean relative reflectance over the 400–700 nm spectral range; and (3) Carotenoid chroma, calculated as (*R*_700_—*R*_450_)/*R*_700_. Hue was manually extracted from the spectrometric data, while mean brightness and carotenoid chroma were computed using the ‘summary()’ function of the *pavo* R package (Maia et al. 2019).

### 2.3 Carotenoid Pigment Analysis

#### 2.3.1 Pigment Extraction

We individually washed each feather in a solution of 1% Triton X‐100, a non‐ionic detergent. Once dried, we weighed the whole feather to the closest hundredth of a milligramme using a Denver Instruments Pinnacle Series PI–225D precision analytical balance (Denver Instruments, Arvada, Colorado, USA), then just the brightly coloured section (pigmented part) of each feather after excision (0.34 to 2.83 mg). We extracted the feather carotenoids of the excised section in warm pyridine in sealed ½ dram borosilicate glass vials flushed with nitrogen gas with the help of a Thermo Scientific Digital Shaking Drybath (Thermo Fisher Scientific, Marietta, Ohio, USA) set at 95°C for 75 min. The extracted carotenoids were subsequently transferred to methyl, *t*‐butyl ether (MTBE) using deionised water. We replaced the deionised water twice to remove as much of the pyridine from the organic epiphase as possible before transferring the MTBE phase with the carotenoids to a clean vial, adding anhydrous sodium sulphate powder to remove any trace of water. The ether extract was evaporated to dryness under a stream of nitrogen (N_2_) and the pigments redissolved in 300 μL of *n*‐hexane (VWR Chemicals BDH 24575). We used this solution to measure the total carotenoid content of the extract. To accomplish this, we fitted the spectrometer with a CUV‐FL‐DA cuvette holder (Ocean Optics) capable of holding 1 cm cuvettes to measure absorbance at the peak of absorption (*λ*_max_). For high‐performance liquid chromatography (HPLC) analysis, we dried the pigment extracts under a stream of nitrogen, redissolved them in 200 μL of HPLC mobile phase (composition noted below) and filtered the resulting solutions using a Millex‐FH PTFE filter, 0.45 μm pore size (Millipore Corp, Bedford, MA) into autosampler vials.

#### 2.3.2 Pigment Identification and Quantification

We quantified individual carotenoids by HPLC using a Waters Alliance HPLC system equipped with an e2695 separations module with a column heater (set at 40°C) and a Waters 2998 photodiode array detector (Waters Corporation, Milford, MA). We separated the carotenoid pigments using a normal‐phase Phenomenex Luna silica column (150 mm × 4.6 mm; 3 μm particle size, 100 Å pore size; Phenomenex, Torrance, California, USA) and an isocratic solvent system of hexane, acetone and acetonitrile (87:11:2 mixture). The elution of carotenoids from the column was monitored at 450 nm. We determined the areas under the curve of individual pigments at that wavelength using the Waters Empower 3 chromatography software.

Carotenoid pigments were identified based on retention times on HPLC, and comparison to reference carotenoids from well described systems that were run alongside flameback samples. We confirmed the pigment identifications, as well as probable functional groups of carotenoids lacking standards, with the help of the UV–visible spectra (300–600 nm) generated by the diode‐array detector. Our references included the carotenoids in the flight feathers of the Yellow‐shafted (β‐carotene, β‐cryptoxanthin, 3′‐dehydro‐lutein, zeaxanthin and lutein) and the Red‐shafted (canthaxanthin, adonirubin, astaxanthin and α‐doradexanthin) forms of the Northern Flicker ( *Colaptes auratus* ; Hudon et al. 2015), the yellow breast feathers of the Western Tanager ( *Piranga ludoviciana* ; canary‐xanthophyll A and B; Hudon 1991) and the orange tail feathers of the American Redstart ( *Setophaga ruticilla* ; papilioerythrinone; Hudon et al. 2025).

#### 2.3.3 Functional Group Composition

**FIGURE 2:**
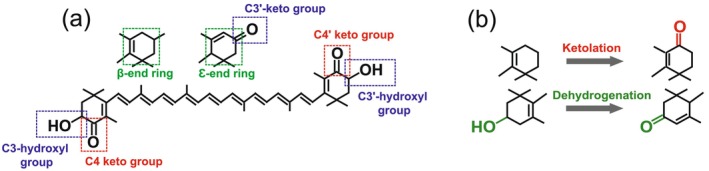
Structural modifications of carotenoids. (a) Illustration of C3(3′)‐oxygenated groups (blue), C4(4′) keto groups (red), β‐ and ε‐end rings (green) in a carotenoid molecule. (b) Changes in molecular structure during ketolation (red) and dehydrogenation (green) enzymatic reactions.

We calculated the prevalence in each carotenoid extract of three simple and largely independent modifications of the β‐carotene backbone that underlie much of the diversity of carotenoids found in most birds (Hudon et al. 2015), including flameback woodpeckers. These modifications are the oxygenation of carbon C4(4′) to yield keto groups (C4(4′)‐keto groups), the oxygenation of carbon C3(3′) to yield hydroxyl or keto groups (C3(3′)‐oxygenated groups) and the conversion of β‐end rings to ε‐end rings (number of ε‐end rings) (Figure 2a). For each structural element, we can derive from the individual pigment concentrations the average extent of that element using the formula: $\bar{a}=\frac{\sum_{i=1}^{n}a_{i}C_{i}}{\sum_{i=1}^{n}C_{i}}$, where *a*_*i*_ is the number of a particular functional group (separately, C4(4′)‐keto groups, C3(3′)‐oxygenated groups or number of ε‐rings) in a specific carotenoid *i* and *C*_*i*_ is the concentration of that pigment in the pigment extract, for all *n* identified carotenoids detected in each feather.

### 2.4 Genome‐Wide Association Study (GWAS)

To identify genomic regions associated with carotenoid colour expression in *Dinopium* flamebacks, we conducted a GWAS analysis using reduced‐representation genomic sequencing of 36 yellow‐backed *D. benghalense* , 45 red‐backed *D. psarodes* , and 25 orange‐backed intermediate flamebacks. The sampling locations of individuals included in the analysis are shown in Figure 1. Detailed methods for DNA extraction, Genotyping‐by‐Sequencing (GBS; Elshire et al. 2011) library preparation, GBS sequencing and subsequent data analysis are provided in Ranasinghe et al. (2024). Briefly, we extracted DNA from blood samples preserved in Queen's lysis buffer using a phenol‐chloroform DNA extraction method. GBS libraries were prepared by digesting genomic DNA with the *PstI* restriction enzyme, followed by ligation of sample‐specific adapters. The DNA fragments were then purified using AMPure XP beads, PCR‐amplified, pooled and size‐selected (400–500 bp) before sequencing. The libraries were sequenced using 150 bp paired‐end reads on Illumina HiSeq 4000 and NovaSeq SP6000 platforms. Sequence data covered approximately 3.7% of the 1.19 Gb *Dryobates pubescens* reference genome (44,246,048 bp; ~294,974 reads; bDryPub1.pri; Vertebrate Genomes Project 2020). The estimated average gap size between reads was approximately 3,877 bp, substantially smaller than typical linkage disequilibrium (LD) block lengths reported in birds (100–400 kb; e.g., Backström et al. 2006; Stapley et al. 2010; Qanbari et al. 2010), ensuring capture of a representative set of tagging SNPs across the Flameback genome. The raw GBS sequences were demultiplexed with a custom Perl script provided in Irwin et al. (2018), trimmed with Trimmomatic 0.32 (Bolger et al. 2014), and aligned to the Downy Woodpecker ( *Dryobates pubescens* ) reference genome (NCBI Assembly: GCA_014839835.1; https://www.ncbi.nlm.nih.gov/datasets/genome/GCF_014839835.1/) with BWA‐MEM using the default parameters. On average, 91.4% of reads were successfully aligned to the reference genome. The genome‐wide single nucleotide polymorphisms (SNPs) data were generated by genotyping and identifying SNPs using GATK 3.8 (McKenna et al. 2010). Variant data were filtered using VCFtools to retain only biallelic SNPs (‐‐min‐alleles 2 and –max‐alleles 2), excluding insertions and deletions (‐‐remove‐indels), retaining sites with a minor allele count ≥ 3 (‐‐mac 3), and ensuring genotypes had a minimum depth of 3 (‐‐minDP 3) and a quality score ≥ 25 (‐‐minGQ 25) (Danecek et al. 2011). We excluded sites with more than 60% missing data (‐‐max‐missing 0.4) and imputed the remaining missing genotypes using BIMBAM 1.0 (Scheet and Stephens 2006), utilising LD information to infer missing data. We estimated a relatedness matrix from the imputed genotypes using GEMMA 0.98.1 (Zhou and Stephens 2012) by calculating the centred relatedness matrix.

To quantify the mantle colouration of flamebacks, we measured luminosity (*L*)—which ranges from black (0) to white (100), a colour matrix (*a*)—spanning from green (negative) to red (positive), and a second colour matrix (*b*)—ranging from blue (negative) to yellow (positive). These measurements were extracted in the LAB colour space using Adobe Photoshop CC (2022) from field photographs of each bird, following the method described by Wang et al. (2020). To account for variation in ambient lighting across images, we standardised all photographs using the white‐balance tool in Adobe Photoshop, referencing a circular white patch naturally present on the wing feathers of each bird as an internal white standard. Colour values (*L*, *a,* and *b*) were obtained from a consistent region on the mantle, covering an area of approximately 3–4 cm^2^. Pixel values within this region were averaged to derive a representative measure of mantle colouration. We then performed a Principal Component Analysis (PCA) on the *L*, *a,* and *b* values. We used the principal component (PC) that explained the most variation in mantle colouration from red to yellow as the phenotypic score for the GWAS analysis (Figure S1). We carried out the GWAS by testing the association between each genetic marker and the mantle colour phenotypic score using GEMMA 0.98.1 with a univariate linear mixed model applying the Wald test (‐lmm 1) (Zhou and Stephens 2012). The resulting data were then visualised using R (R Core Team 2024).

#### 2.4.1 Read Depth Analysis

Genomic regions that involve duplications or Copy Number Variations (CNVs) can be detected by analysing the read depth and allelic ratios of a particular genomic region (Abel and Duncavage 2013; Kadalayil et al. 2015; Karunarathne et al. 2023; Zhao et al. 2013). Increased read depth with a 3:1 allele ratio would indicate the possibility of a duplication event. Very high increased read depths would indicate the possibility of many such duplications. Hence, to further examine SNPs that indicated possible signs of multiple copies, we calculated the allele depths (reference and alternative allele depths) using bcftools (Danecek et al. 2021) and visualised the results in R using the *ggplot2* package (R Core Team 2024; Wickham and Sievert 2016).

### 2.5 Statistical Analysis

We conducted all statistical tests in R (R Core Team 2024). For all statistical hypothesis tests, we calculated the *p* value adjusted for false discovery rate (FDR) and considered 0.05 as the *p* value significance threshold. When comparing various colour and carotenoid metrics—including mean hue, mean brightness, mean carotenoid chroma, mean concentrations of different pigments and mean number of functional groups of carotenoids—among the three phenotypic groups, we performed Fisher–Pitman permutation tests using the *coin* R package with 1,000,000 re‐samplings (Hothorn et al. 2008). In instances where the permutation test indicated significant differences among groups, post hoc analyses were conducted using the *rcompanion* R package (Mangiafico 2024) to perform pairwise tests and identify specific pairs of groups with significant differences. Additionally, Spearman's rank correlation tests were employed to analyse correlations between mantle hue and each functional group composition variable, including C4(4′)‐keto groups, C3(3′)‐oxygenated groups and the number of ε‐end rings.

## 3 Results

### 3.1 Reflectance Spectrometry and Colour Metrics

**FIGURE 3:**
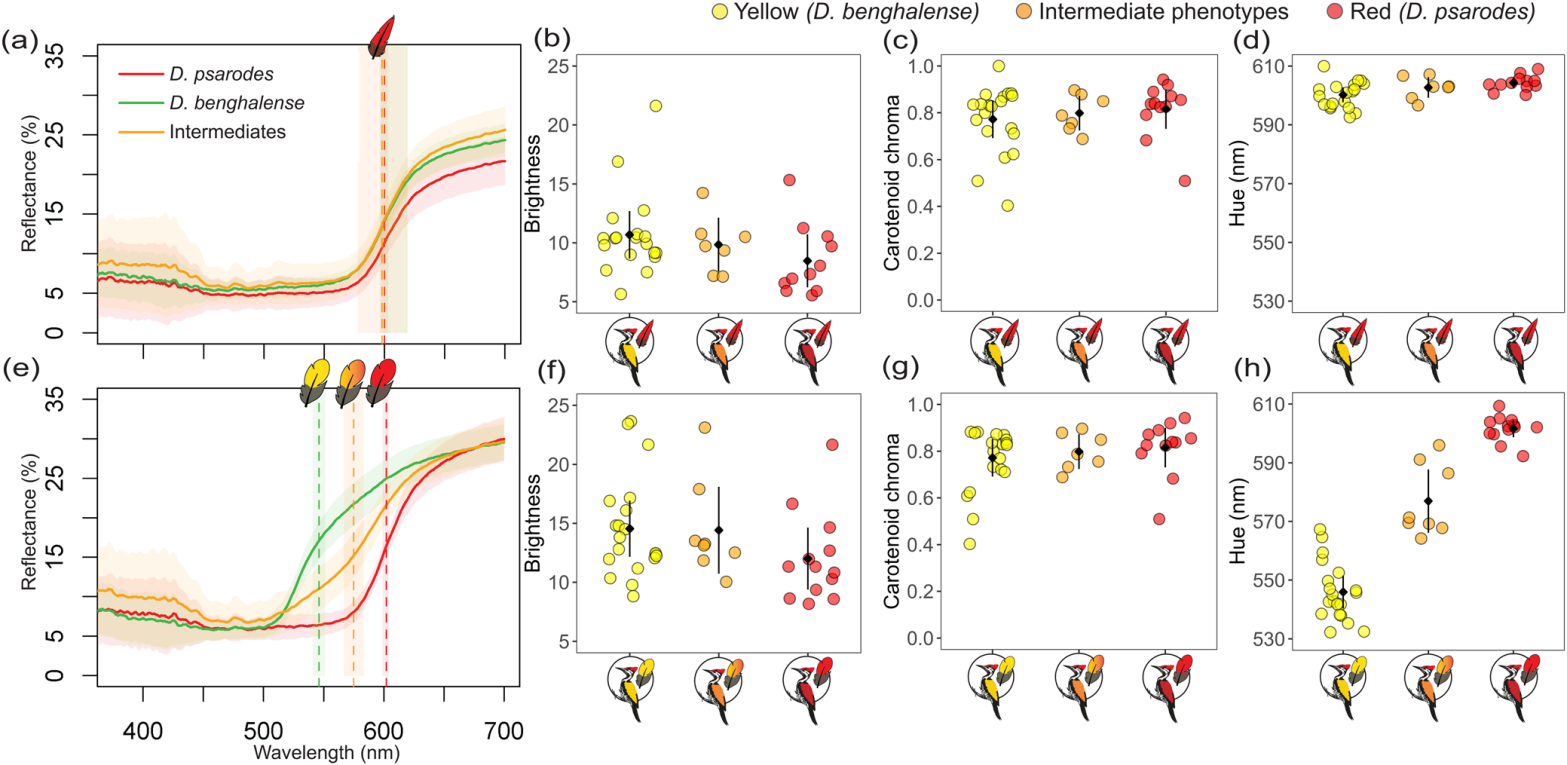
Reflectance curves and colorimetric variables for crown (top) and mantle (bottom) feathers across three phenotypic groups: Yellow‐backed *D. benghalense* (yellow), orange‐backed intermediates (orange) and red‐backed *D. psarodes* (red). Panels (a) and (e) show reflectance curves for crown and mantle feathers, respectively, with solid lines representing the mean reflectance curves and shaded areas indicating the 95% CI of the mean. Dashed vertical lines denote the mean hue (*λ*_R50_) of each reflectance curve, with the shaded areas representing the 95% CI of the mean hue (*λ*_R50_). Panels (b) and (f) display mean brightness, (c) and (g) the carotenoid chroma and (d) and (h) the mean hue (*λ*_R50_) for crown and mantle feathers, respectively. In these plots black diamonds represent the estimated population mean, and error bars indicate the 95% CI of the mean.

We present reflectance data for the three phenotypic groups in Figure 3. The mantle feathers of the three phenotypic groups differed significantly in hue ($\lambda_{R50}$) (permutation test: *p* < 0.05) (Figure 3e,h). The yellow mantle feathers of *D. benghalense* had the lowest mean value of $\lambda_{R50}$ at 546 nm (95% CI: 541–551 nm), while the red mantle feathers of *D. psarodes* had the highest at 602 nm (95% CI: 599–604 nm). Orange‐coloured mantle feathers of intermediate birds had mean $\lambda_{R50}$ values in between those of red and yellow feathers (mean hue = 575 nm; 95% CI: 567–583 nm). The crown feathers of all groups exhibited a mean $\lambda_{R50}$ value at long wavelengths, consistent with their red hue ( *D. psarodes* —mean hue = 600 nm, 95% CI: 597–603 nm; *D. benghalense* —mean hue = 598 nm, 95% CI: 596–600 nm; intermediate birds—mean hue = 598 nm, 95% CI: 578–619 nm) (Figure 3a,d).

In contrast, the mean brightness and carotenoid chroma of mantle feathers did not differ significantly among the groups (Figure 3f,g). Also, we obtained similar colour metric values for hue, mean brightness and carotenoid chroma for the crown feathers of birds across all phenotypic groups, with no significant difference among them (permutation test: *p* value > 0.05; Figure 3b–d).

### 3.2 Carotenoid Pigment and Functional Group Variability

**FIGURE 4:**
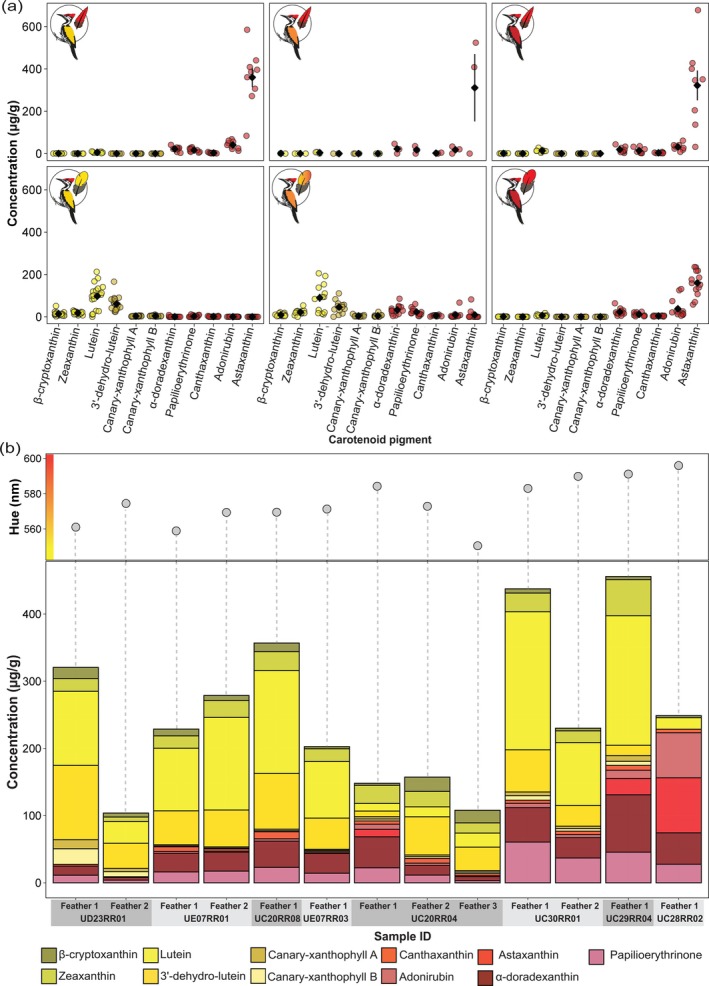
Carotenoid pigment concentrations. (a) Concentration of carotenoids (light yellow: Dietary yellow carotenoids, dark yellow: Metabolised yellow carotenoids, red: Metabolised red carotenoids) in crown (top) and mantle (bottom) feathers of the three phenotypic groups: Yellow‐backed *D. benghalense* (left), intermediate‐plumaged flamebacks (middle) and red‐backed *D. psarodes* (right). The black diamond shows the estimated population mean, and the error bar the 95% CI of the mean. (b) Absolute concentration and composition of red and yellow carotenoid pigments in the mantle feathers of all intermediate orange‐backed flamebacks including those for which multiple feathers were analysed. The scatter plots at the top display the hue (*λ*_R50_) for each mantle feather.

The red mantle feathers of *D. psarodes* contained predominantly astaxanthin, along with small amounts of adonirubin, α‐doradexanthin, papilioerythrinone and canthaxanthin (Figure 4a‐bottom row of plots, Figures S2 and S3). The red carotenoids were accompanied by trace amounts of the dietary yellow carotenoid lutein (Figures 4a and S3). In contrast, the yellow mantle feathers of *D. benghalense* contained mainly lutein and metabolised 3′‐dehydro‐lutein, along with small amounts of zeaxanthin and β‐cryptoxanthin and metabolised yellow carotenoids canary‐xanthophyll A and canary‐xanthophyll B (Figures 4a, S2 and S3). Additionally, yellow feathers contained minimal amounts of red carotenoids including papilioerythrinone, α‐doradexanthin and canthaxanthin (Figures 4a and S2). The orange feathers of birds with intermediate plumage colours harboured both the red and yellow pigments found in the mantle feathers of *D. psarodes* and *D. benghalense* , but in varying proportions (Figures 4a,b, S2 and S3). In most of the birds of intermediate colour, the red colouration resulted from α‐doradexanthin and papilioerythrinone, rather than from astaxanthin, the predominant carotenoid in *D. psarodes* (Figures 4b and S3). Shades of orange in intermediate‐coloured feathers resulted from varying blends of red and yellow carotenoid pigments. We found that feathers of different orange hues from single individuals also differed in composition and relative concentration of their carotenoids (Figures 4b and S3). The red crown feathers of all three phenotypes contained a similar suite of carotenoids to those identified in the mantle feathers of *D. psarodes* (Figure 3a‐top row of plots, Figures S2 and S3). However, the astaxanthin concentration was substantially higher in crown feathers compared to mantle feathers (Figures 3a and S3).

**FIGURE 5:**
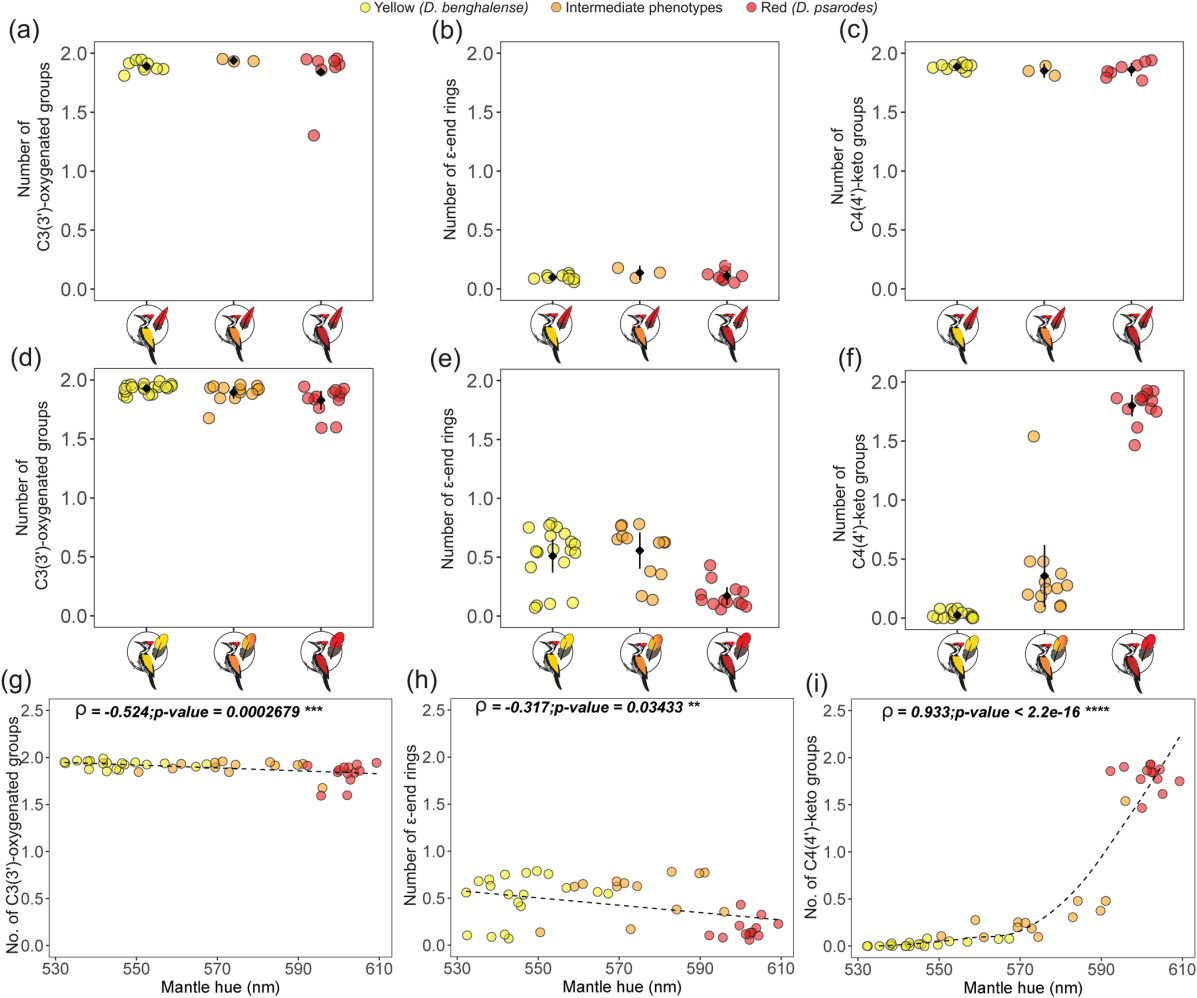
Frequency of functional groups and their correlation with mantle hue. (a) Average number of C3(3′)‐oxygenated groups, (b) number of ε‐end rings and (c) number of C4(4′)‐keto groups of aggregate of all carotenoids in crown feathers for the three phenotypic groups. (d) Average number of C3(3′)‐oxygenated groups, (e) number of ε‐end rings and (f) number of C4(4′)‐keto groups of aggregate of all carotenoids in mantle feathers for the three phenotypic groups. Black diamonds represent the estimated population mean, and the error bars represent the 95% CI of the means. Panels (g–i) show the relationship between mantle hue and (g) C3(3′)‐oxygenated groups, (h) ε‐end rings and (i) C4(4′)‐keto groups. The Spearman's rank correlation coefficient (*ρ*) and the *p* values from the Spearman's rank correlation hypotheses test are shown in each correlation plot. Dashed lines indicate the regression lines, provided for visualisation of the correlations. Colours correspond to the phenotype of the bird: Yellow for yellow‐backed *D. benghalense* , orange for intermediate‐plumaged hybrid flamebacks, and red for red‐backed *D. psarodes* .

The carotenoids in the mantle feathers of different phenotypic groups differed significantly in the types of functional groups they harboured (permutation test: *p* value < 0.002; Table S1). The average number of oxygenated groups (hydroxyl or keto) at C3(3′) on the carotenoids in *D. psarodes* was significantly lower than that in *D. benghalense* (permutation test: *p* value = 0.002077; post hoc analysis with pairwise tests: *p*‐adjusted = 0.0067), while it did not differ significantly between *D. benghalense* and intermediate birds, and between *D. psarodes* and intermediates (Figure 5d; Tables S1 and S2). The carotenoids in the red mantle feathers of *D. psarodes* exhibited significantly fewer ε‐end rings compared to those in *D. benghalense* and the birds with an intermediate (orange) phenotype (permutation test: *p* value = 0.000044; Figure 5e; Tables S1 and S2). The frequency of ε‐end rings did not differ significantly between *D. benghalense* and the intermediate‐coloured birds (Figure 5e; Table S2). Lastly, the average number of C4(4′)‐keto groups differed significantly among all three phenotypic groups (permutation test: *p* value < 0.0001). Specifically, *D. psarodes* had the highest frequency of C4(4′)‐keto groups, followed by intermediate birds, with *D. benghalense* having the lowest (Figure 5f; Table S1 & S2). Crown feathers across all phenotypic groups exhibited elevated levels of C3(3′)‐oxygenated and C4(4′)‐keto functional groups, along with reduced levels of ε‐end rings (Figure 5a‐c), closely resembling the functional group composition observed in the red mantle feathers of *D. psarodes* . None of the functional group variables differed significantly among the phenotypic groups (permutation test: *p* > 0.3; Table S1), indicating a similar functional group profile in crown feathers across all groups.

Mantle hue ($\lambda_{R50}$) correlated significantly with the functional group metrics we assessed (Figure 5g–i). The average number of C3(3′)‐oxygenated groups (Spearman's rank correlation coefficient (*ρ*) = −0.524) and ε‐end rings (*ρ* = −0.317) decreased significantly when moving from yellow to red hues (Spearman's rank correlation; *p* value < 0.05) indicating that higher levels of these functional groups characterise the yellow phenotype, while lower levels characterise the red phenotype (Figure 5g,h). In contrast, the number of C4(4′)‐keto groups increased significantly with redness (Spearman's rank correlation; *p* value < 2.2e‐16, *ρ* = 0.933) highlighting a strong association between C4(4′)‐ketolation and red colouration (Figure 5i).

### 3.3 Genomic Architecture of Carotenoid Colouration

**FIGURE 6:**
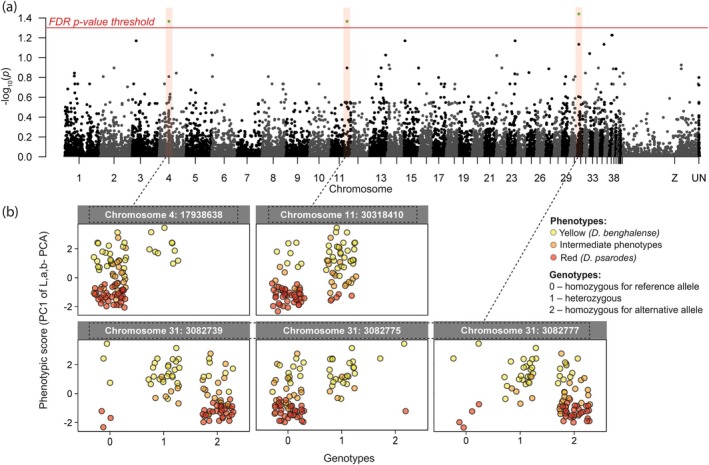
GWAS Manhattan plot and genotypes of SNPs significantly associated with mantle colour variations in *Dinopium* flamebacks. (a) Manhattan plot for the GWAS analysis of *Dinopium* flameback mantle feather colouration. Log‐transformed *p* values, adjusted for false discovery rate are plotted against chromosomal positions. The red solid line indicates the genome‐wide significance threshold (−log_10_(0.05)). Please note that on chromosome 4, four SNPs appear as a single point due to identical −log_10_(*p*) exceeding the threshold and similarly, seven SNPs on chromosome 31. (b) Strip charts showing the phenotypic scores of *Dinopium* flamebacks against genotypes for SNPs significantly associated with carotenoid colour expression. For chromosome 4, only one representative SNP is shown due to similar genotype patterns across four adjacent SNPs. For chromosome 31, three of the seven significant SNPs are shown as representative examples. Colours denote different phenotypic groups: Yellow for yellow‐backed *D. benghalense* , orange for intermediate‐plumaged flamebacks and red for red‐backed *D. psarodes* .

The GWAS analysis revealed that mantle carotenoid colouration in *Dinopium* flamebacks is associated with 12 SNPs distributed across three genomic regions: four SNPs on chromosome 4, one SNP on chromosome 11, and seven SNPs on chromosome 31 (Figure 6a). We note that all 4 SNPs on chromosome 4, as well as the 7 SNPs on chromosome 31, have the same −log_10_ (*p*), therefore each group appears as a single point on the Manhattan plot (Figure 6a). To further evaluate the association of these regions with carotenoid colouration, we calculated windowed *F*_ST_ following the methodology detailed in Irwin et al. (2018). Briefly, *F*_ST_ values were computed across non‐overlapping windows of 10,000 sequenced base pairs genome‐wide to quantify genetic differentiation between allopatric red‐backed ( *D. psarodes* ) and yellow‐backed ( *D. benghalense* ) phenotypic groups based on differences in allele frequencies (Figure S4). This analysis revealed *F*_ST_ peaks on chromosomes 4, 11 and 31, which co‐localise with regions identified in the GWAS. The concordance between GWAS signals and *F*_ST_ peaks provides additional support for these regions to be candidates underlying carotenoid‐based colouration differences in *Dinopium* flamebacks.

The SNPs on chromosome 4 are located within the *PIGQ* gene, which encodes a protein involved in glycosylphosphatidylinositol (GPI)‐anchor biosynthesis (Nakamura et al. 2012; Wu et al. 2020). The GPI‐anchor plays a crucial role in anchoring proteins to the cell surfaces of many blood cells. The SNP on chromosome 11 was situated in an exon region of the *CYP2J2* gene (Figure S5), a candidate gene for carotenoid ketolation. The seven SNPs on chromosome 31 were located within an intron region of the *HSD11B1L* gene (Figure S5), which catalyses the interconversion of inactive cortisone to corticosterone (the avian stress hormone). To determine whether any nearby genes might be the actual causal loci due to linkage, we examined genes within a ±20,000 bp window around each significant SNP for previously reported associations with carotenoid colour expression. On chromosome 4 and 31, none of the adjacent genes demonstrated any known association with carotenoid expression. However, on chromosome 11, we identified two additional copies of *CYP2J2*‐like genes flanking the SNP in the *CYP2J2* gene, resulting in a total of three *CYP2J2*‐like genes present in the reference genome on chromosome 11. Genes involved in the ketolation step of carotenoid metabolism, such as *CYP2J19* and *CYP2J40* are variably annotated as *CYP2J2* or *CYP2J2*‐like across most genomes (Mundy et al. 2016). To clarify the identity of the *CYP2J2* locus in the Downy Woodpecker ( *Dryobates pubescens* ) reference genome, we conducted a BLAST search against the Zebra Finch ( *Taeniopygia guttata* ; bTaeGut7.mat) reference genome, in which *CYP2J19* and *CYP2J40* have been well characterised. The query sequence aligned to three distinct regions of the *CYP2J40* gene on chromosome 8 of the Zebra Finch genome, with an approximate query coverage of 19%, suggesting a closer homology to *CYP2J40*.

Examination of genotypes for these SNPs indicated that most flamebacks with red and intermediate colour plumages are homozygous for these SNPs (Figure 6b and S6). Notably, SNPs on chromosomes 4 and 11 displayed a genomic pattern where the majority of birds were called homozygous for the reference allele, with some being called heterozygous, and no birds were called homozygous for the non‐reference (alternative) allele (Figure 6b). This genotype pattern could indicate the presence of multiple copies of these genomic regions, with most copies likely homozygous for the reference allele. Another plausible explanation for this pattern is that the alternative allele may be genuinely rare within the population, leading to a lack of individuals homozygous for the alternative allele in our dataset.

Read depth analysis indicated a significant elevation in read depths for the outlier SNPs located on chromosomes 4 and 11, surpassing the genome‐wide average read depth for each individual. Notably, certain individuals exhibited read depths exceeding 22 times the mean genome‐wide level (Figure S7; representative raw read alignments from individuals [*n* = 2] across each phenotypic group are provided in Figures S8–S10). These elevated read depths may indicate the potential presence of multiple copies of these genomic regions. Specifically, for the SNP on chromosome 4, individuals within the red and intermediate phenotypic groups exclusively carried the reference allele, implying potential duplication of this region, which may be homozygous for the reference allele (Figure S7). Similarly, the SNP on chromosome 11 demonstrated elevated read depth patterns solely within the red phenotypic group. These observations suggest that the allele composition across multiple copies of these genomic regions may vary between species. Increased within‐species variation in read depths could further suggest the existence of copy number variations even within individual populations. We note, however, that there is a possibility that amplification biases related to GC content or fragment length may contribute to the elevated read depths for the loci in question (DaCosta and Sorenson 2014). Additional analyses are required to elucidate the architecture of these copy number variations and their precise influence on carotenoid colour expression in *Dinopium* flamebacks.

## 4 Discussion

As expected from the difference in mantle feather colouration of the two *Dinopium* species we studied, there were marked differences in the profile of carotenoid pigments harboured by the feathers. The red mantle feathers of *D. psarodes* harboured primarily astaxanthin along with small amounts of adonirubin, α‐doradexanthin, papilioerythrinone and canthaxanthin, all red carotenoids with keto groups at C4(4′). In contrast, the yellow mantle feathers of *D. benghalense* harboured predominantly yellow carotenoids, primarily dietary carotenoid lutein and metabolised 3′‐dehydro‐lutein along with minor amounts of dietary (zeaxanthin and β‐cryptoxanthin) and metabolised (canary‐xanthophylls A and B) carotenoids. The hybrids with intermediate‐coloured (orange) mantle feathers exhibited a combination of red and yellow pigments, featuring the same pigments as in the two parental forms, but in different proportions.

The suite of carotenoids used by hybridising *Dinopium* flamebacks in Sri Lanka is not unlike that described in other woodpecker lineages, such as the Red‐shafted ( *Colaptes auratus cafer* ) and Yellow‐shafted (*C. a. auratus*) forms of the Northern Flicker in North America (Hudon et al. 2015), another conspicuously‐coloured woodpecker taxon with distinct red and yellow forms that hybridise in the wild. In that system, the yellow flight feathers of the eastern *C. a. auratus* harbour yellow carotenoids that for the most part can be acquired directly in the diet—primarily lutein, along with some β‐cryptoxanthin, zeaxanthin, 3′‐dehydro‐lutein and β‐carotene—while the red flight feathers of western *C. a. cafer* harbour predominantly red 4‐keto‐carotenoids—adonirubin, α‐doradexanthin, canthaxanthin and astaxanthin, representing ketolated products of the yellow pigments in the yellow‐shafted form (Hudon et al. 2015). We note that in *C. a. cafer*, adonirubin was the dominant keto‐carotenoid in the flight feathers, while astaxanthin, which featured predominantly in the red feathers of *Dinopium* flamebacks, was more abundant in the malar stripe and nuchal patch of the species. Additionally, Stradi et al. (1998) reported that, in a study of multiple woodpecker lineages, astaxanthin or another 4‐keto‐carotenoid, α‐doradexanthin, are the primary carotenoids found in the crown feathers of species across these lineages, typically accompanied by smaller amounts of adonirubin and canthaxanthin. Furthermore, in contrast to the red feathers, the yellow feathers of woodpeckers predominantly harbour lutein, zeaxanthin and β‐cryptoxanthin, while greenish or light yellow feathers in a few species also harbour carotenoids with shortened chains of double bonds known as picofulvins—carotenoid pigments only known from woodpeckers (Stradi et al. 1998). Picofulvin‐like pigments (with blue‐shifted absorption spectra) were not conspicuous contributors to the yellow colour of the mantle feathers of *D. benghalense* .

None of the previous studies of carotenoids in woodpeckers (Hudon et al. 2015; Stradi et al. 1998) documented three particular carotenoids we found in *Dinopium* flamebacks, all three bearing ε‐end rings. These include the two canary‐xanthophylls (canary‐xanthophyll A and B), metabolised carotenoids with two ε‐end rings that we found in the yellow feathers of *D. benghalense* and orange‐backed hybrid flamebacks. These carotenoids have been reported in the yellow feathers of other avian lineages such as canaries (Koch et al. 2016), cardinalids (Hudon 1991; McGraw et al. 2003), finches (Ligon et al. 2016; McGraw et al. 2006; McGraw and Gregory 2004), cotingas (Prum et al. 2012), widowbirds (Andersson et al. 2007) and caciques and meadowlarks (Friedman et al. 2014). The other carotenoid is papilioerythrinone, a mono‐4‐keto‐carotenoid with a ketolated ε‐end ring found in the two *Dinopium* species and their hybrids. Papilioerythrinone has so far been reported in only a few species with red feathers or integuments; for example, the feathers of the male American Redstart ( *Setophaga ruticilla* ; Hudon et al. 2025), male Bullfinch ( *Pyrrhula pyrrhula* ; Stradi et al. 2001), Gouldian finch ( *Erythrura gouldiae* ; Toomey et al. 2018), Black‐and‐Crimson Oriole ( *Oriolus cruentus* ; LaFountain et al. 2013), and the integument of the Red‐legged Partridge ( *Alectoris rufa* ; García‐de Blas et al. 2014).

The differences in pigment composition between *D. psarodes* and *D. benghalense* (and their hybrids) largely explain the variation in mantle feather colouration observed across the different phenotypic groups. The carotenoids in *D. psarodes* as an aggregate had a high average number of keto groups at C4(4′), whereas those in *D. benghalense* exhibited a high average number of ε‐end rings, while those in the orange‐backed hybrid flamebacks had intermediate levels of these functional groups. Keto groups at C4(4′) elongate the carotenoids' conjugated double‐bond system, which shifts the absorbance peak to longer wavelengths, resulting in a redder colour, whereas ε‐end rings, as in lutein and the metabolised carotenoids canary‐xanthophylls A and B, shorten that system, shifting absorbance to shorter wavelengths, resulting in yellower colours (Brush 1990; McGraw 2006; Stradi 1998). The shade of orange observed in the orange‐backed hybrid birds also correlated with the frequency of these functional groups (Figure 4b and S3). It was not uncommon for birds with orange‐coloured mantles (hybrids) to have feathers displaying different hues (redness), which we also found to be correlated with pigment and functional group composition and relative concentration (Figure 4b and S3). The hue also varied as a function of a chemical modification, the oxygenation (which includes hydroxylation) at C3(3′) that is not expected to affect colour. However, hydroxylation at C3(3′) may be needed to produce ε‐end rings, which in turn will affect the number of keto groups that can be added at the C4 position of the molecule, as ε‐end rings do not permit this modification (Hudon et al. 2015, 2025).

The types of carotenoids observed in the *Dinopium* flamebacks were similar between *D. psarodes* and *D. benghalense* as well as their hybrids, which indicate that both species are able to perform ketolation (Figure 2b) at C4(4′) position and the conversion of β‐end rings into ε‐end rings (and possibly vice versa) through dehydrogenation (Figure 2b; Prum et al. 2012). Indeed, we found small quantities of the yellow dietary carotenoid lutein in the red feathers of *D. psarodes* , as well as small amounts of red 4‐keto‐carotenoids (canthaxanthin and α‐doradexanthin) in the yellow feathers of *D. benghalense* . Moreover, both species deposited the same suite of 4‐keto‐carotenoids, including papilioerythrinone, in their red crown feathers. All red keto‐carotenoids found in *D. psarodes* result from ketolation of various precursors at C4(4′) (García‐de Blas et al. 2014; Stradi 1998). Similarly, *D. benghalense* uses ketolation to produce the red pigments in its crown feathers, and also the observed red 4‐keto‐carotenoids in its yellow mantle feathers. In addition, both species can carry out the dehydrogenation of dietary or metabolised carotenoids to produce pigments with ε‐end rings, notably the two canary‐xanthophylls in *D. benghalense* and papilioerythrinone in the two parental species and hybrids. These observations collectively imply that both species have the enzymatic capabilities to perform the same reactions in both crown and mantle feathers, but do so to varying degrees. Thus, differential expression or regulation of the genes associated with these biochemical pathways may underlie the tissue‐specific differences in carotenoid metabolism observed between the two species.

**FIGURE 7:**
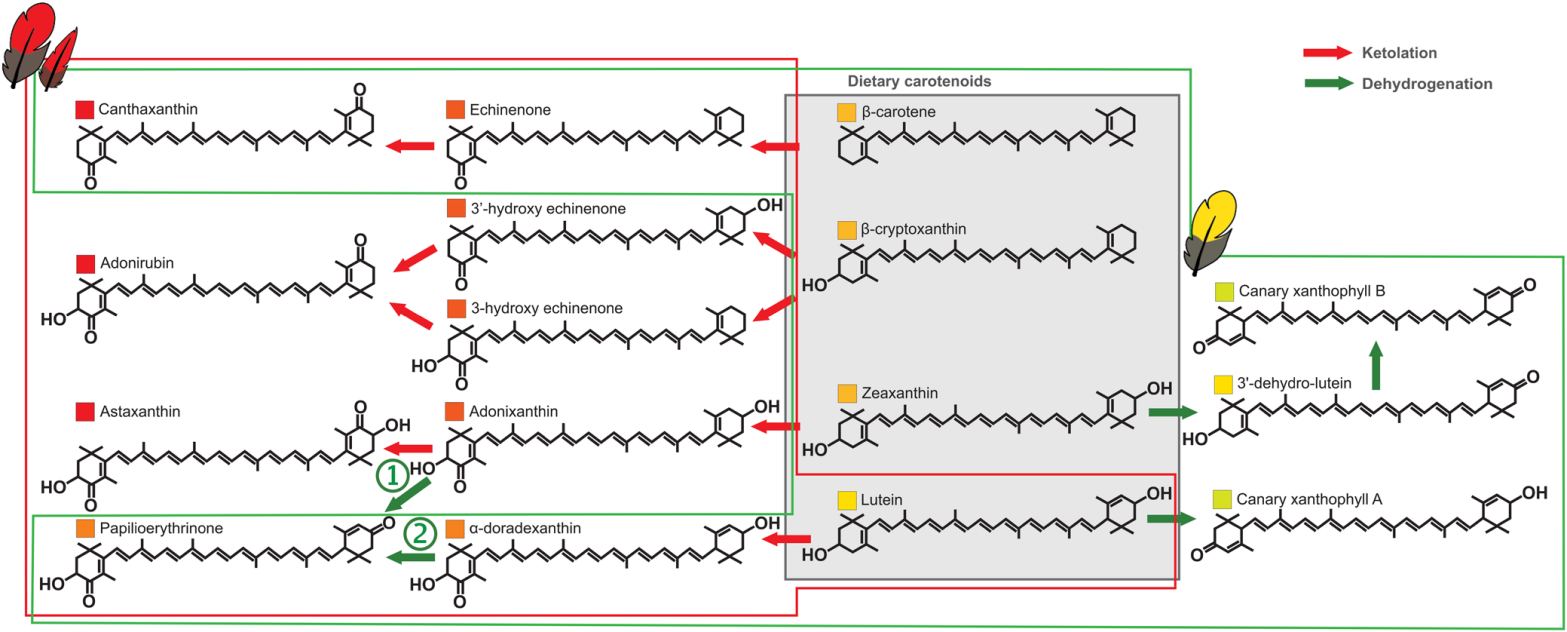
Proposed metabolic pathways of carotenoid pigments identified in the red and yellow mantle feathers of *Dinopium* flamebacks. The numbered arrows indicate two possible pathways leading to the production of papilioerythrinone in flamebacks: (1) the β‐dehydrogenation of the β‐end ring of adonixanthin and (2) the α‐dehydrogenation of the hydroxyl group on the ε‐end ring of α‐doradexanthin.

The difference in relative expression of the enzymatic activities between the two species could help explain why α‐doradexanthin and papilioerythrinone, two pigments with ε‐end rings and a single 4‐keto group, were the predominant 4‐keto‐carotenoids present in most orange‐backed intermediate flamebacks (Figure 4b). This contrasts with the use of astaxanthin, a carotenoid which lacks ε‐end rings and instead possesses two β‐end rings each with a keto group at C4(4′), as the main carotenoid in the mantle feathers of *D. psarodes* and one of the hybrids and the crown feathers of individuals of all phenotypic groups (Figure 4 and S3). Many of the carotenoid pigments found in the mantle feathers of *D. benghalense* (including lutein and 3′‐dehydro‐lutein) feature ε‐end rings, and birds of intermediate colour (hybrids) could inherit a propensity to produce such ε‐end rings from *D. benghalense* . We speculate that in these flameback woodpeckers papilioerythrinone may actually result from the β‐dehydrogenation of the β‐end ring of adonixanthin (arrow 1 in Figure 7) rather than the α‐dehydrogenation of the hydroxyl group on the ε‐end ring of α‐doradexanthin (arrow 2 in Figure 7) suggested for other species where the pigment has been found (García‐de Blas et al. 2014). *D. psarodes* largely avoids the production of carotenoids with ε‐end rings in its mantle, which permits it to produce carotenoids with two keto groups (instead of just one as in α‐doradexanthin). It would be instructive to track the chromosomal location and inheritance of the genetic element that contributes to this difference. The products of two genes have recently been implicated as responsible for the production of 4‐keto groups and ε‐end rings in birds, whose respective activities are closely linked to each other: *CYP2J19* and *BDH1L* (Toomey et al. 2022, 2025). It has been suggested that the product of the *CYP2J19* gene produces an intermediate, likely a 4‐hydroxy‐carotenoid, that when acted upon by the product of the *BDH1L* gene becomes a 4‐keto‐carotenoid. But when *BDH1L* acts alone it results in the production of an ε‐end ring (Toomey et al. 2022). Consequently, we might expect that altering the balance between the two genes would result in the production of different types of carotenoids, some with keto groups, others with ε‐end rings. We note that *BDH1L* is required for both reactions, while *CYP2J19* is only required for the production of 4‐keto carotenoids.

The GWAS admixture analysis identified *PIGQ, CYP2J2* and *HSD11B1L* as genes associated with the carotenoid colour differences observed between the two *Dinopium* species. While *PIGQ* has not been previously linked to carotenoid metabolism, its role in GPI‐anchor biosynthesis could potentially affect cell membrane dynamics and, consequently, carotenoid transport or storage. As mentioned above, the *CYP2J2*‐like gene family is a well‐documented catalyst in the ketolation of dietary carotenoid precursors across various avian lineages, such as zebra finches (Mundy et al. 2016), canaries (Lopes et al. 2016), weaverbirds (Twyman et al. 2018), tinkerbirds (Kirschel et al. 2020), cardinals (Sin et al. 2020), ostriches (Twyman et al. 2016), chickens (Watanabe et al. 2013) and even in non‐avian species such as turtles (Twyman et al. 2016) and fish (Xu et al. 2023). Aguillon et al. (2021) reported an association between the *CYP2J19* gene and red and yellow carotenoid colouration in the flight feathers (wing and tail) of Northern Flickers (*Colaptes auratus*). The *HSD11B1L* gene, documented to be involved in corticosterone regulation, suggests a broader connection between stress response pathways and carotenoid‐based colouration. Interestingly, *HSD11B1* was also identified as a candidate gene in a recent study of carotenoid colouration in House Finches ( *Haemorhous mexicanus* ; Koch et al. 2025). Although the mechanistic role of *HSD11B1* in colouration remains unclear, stress‐induced carotenoid expression has been observed across several vertebrate taxa—including lizards, fish and birds—where elevated corticosterone levels are often associated with increased redness (Costantini et al. 2008; Fairhurst et al. 2015; Kennedy et al. 2013; Lendvai et al. 2013; Loiseau et al. 2008; Romero and Fairhurst 2016). For example, increased corticosterone levels have been linked to elevated circulating carotenoid levels and brighter carotenoid‐based colouration in beaks of mallard ducks (Fairhurst et al. 2015), greater redness in adult male redpolls (Fairhurst et al. 2014) and enhanced plumage redness in male house finches (Lendvai et al. 2013). Conversely, elevated corticosterone levels have been associated with a reduction in carotenoid hue in yellow warblers (Grunst et al. 2015). Similarly, carotenoid colour expression in *Dinopium* may be influenced by stress‐related pathways. Further research is needed to clarify the nature of this relationship and its implications for carotenoid colouration in this species. Moreover, we emphasise that the use of GBS data in this study presents inherent limitations for genome‐wide association analyses, particularly in detecting the full spectrum of genotype–phenotype associations and identifying causal variants with high resolution (Szarmach et al. 2021). As such, while the genes identified provide promising leads, we acknowledge that the reduced representation of the genome in GBS likely constrained our ability to capture the complete genetic architecture of carotenoid colouration in *Dinopium*.

The evidence of variation in allele numbers highlights the complexity of carotenoid colour expression in flamebacks, with the *CYP2J2* gene playing a significant role in determining the carotenoid‐based plumage colourations observed in the *Dinopium* flameback species. The presence of multiple copies of the gene and differences in allele frequency across species suggest that the regulation of carotenoid metabolism and colouration may involve both gene duplication and variation in allele expression. The evolutionary history of *CYP2J2‐*like genes across lineages has shown evidence of loss and duplications (Twyman et al. 2016). Furthermore, the presence of multiple copies of *CYP2J2*‐like genes has been reported in the zebra finch, where wild‐type red‐beaked zebra finches have three *CYP2J2*‐like genes, whereas yellow‐beaked individuals possess only two copies as a result of the deletion of one *CYP2J2*‐like locus (Mundy et al. 2016). These copies exhibit tissue‐specific expression where *CYP2J19A* is expressed in the retina and *CYP2J19B* is expressed in the beak and tarsus (Mundy et al. 2016). Additional research including gene expression analysis (qRT‐PCR) and analysis of long‐read whole genome sequencing (WGS) variant data will be needed to validate and fully understand the nature and role of multiple copies of *CYP2J2* genes in carotenoid colour variation in *Dinopium* flamebacks. Moreover, *CYP2J2* alone may not fully account for variation in carotenoid colour expression in *Dinopium*. It is likely that additional genes are involved in regulating carotenoid colouration, potential candidates being *BDH1L* or an upstream regulatory element. The existence of variation between different feathers of single individuals underscores the complex genetic and biochemical interactions that influence colour expression in hybrid flamebacks. Further study will be needed to determine whether this intra‐individual variability relates to diet, seasonality or body condition at the time of feather moult.

Considering that the red and yellow flameback forms only differed significantly in hue and not in brightness and carotenoid chroma suggests that hue is the most important difference. The variation of hue may reflect specific ecological adaptations, such as habitat preferences or differences in signalling functions under varying light conditions. As noted by Ranasinghe et al. (2024), the red‐backed phenotype of *D. psarodes* appears to be adapted to the wet and humid habitats of the island, while the lighter yellow‐backed *D. benghalense* occurs in the drier, more arid habitats. We hypothesise that *D. psarodes* co‐opted the processes responsible for the red colour of the crown of this genus (and other woodpeckers) to produce red carotenoids also in its mantle feathers, a yellow mantle being the norm in all other *Dinopium* flameback woodpeckers (Fernando 2020). Exploring these ecological contexts further may provide deeper insights into how these colouration patterns contribute to fitness and survival in their respective habitats. This, in turn, could enhance our understanding of the evolutionary dynamics of divergence within the flameback woodpeckers, particularly the role of carotenoid‐based colouration in contributing to reproductive isolation or mate selection. Interestingly, despite differences in mantle colouration, both *Dinopium* species and hybrids exhibit a conserved red pigment composition and hue in their crown feathers. This consistency suggests that the red hue in crown feathers may serve a common functional or signalling role across all lineages, one that is not subject to the same selective pressures as the mantle colouration.

In conclusion, our findings suggest that carotenoid colour expression of *Dinopium* flamebacks is driven by a complex interplay between carotenoid metabolism, stress response pathways and potential regulatory effects on carotenoid expression. The observed differences in mantle colouration, primarily reflected in hue, may highlight species‐specific adaptations to distinct ecological environments, while the conserved red crown colouration across *Dinopium* lineages likely serves a consistent functional or signalling role. The identification of genes such as *CYP2J2*, *HSD11B1L* and *PIGQ* through GWAS admixture analysis underscores the complex role of genetic factors in carotenoid bioconversion and colour expression in these birds. The allelic variation and the evidence of the presence of multiple copies of the *CYP2J2‐*like gene further suggest a complex genetic regulation of carotenoid colour expression in *Dinopium* flamebacks. Collectively, these insights enhance our understanding of the genetic and biochemical mechanisms driving carotenoid colour variation in *Dinopium* species and set the stage for future research into the evolutionary and ecological significance of these traits.

## Author Contributions

This study was conceived of primarily by Rashika W. Ranasinghe, with contributions from all authors. Rashika W. Ranasinghe and Sampath S. Seneviratne designed and conducted the field sampling. Rashika W. Ranasinghe and Jocelyn Hudon carried out carotenoid pigment extractions and HPLC chromatography. Jocelyn Hudon analysed the HPLC data. Rashika W. Ranasinghe conducted the DNA extraction, GBS library preparation, genomic and pigment data analysis and data visualisation, under significant guidance and supervision of Darren Irwin. The manuscript was drafted by Rashika W. Ranasinghe and Jocelyn Hudon, with substantial contributions and revisions from Darren Irwin and Sampath S. Seneviratne.

## Conflicts of Interest

The authors declare no conflicts of interest.

## Supporting information


**Data S1:** mec70084‐sup‐0001‐Supinfo01.pdf.

## Data Availability

Data processing and visualisation scripts along with the associated metadata are available in the Dryad repository (https://doi.org/10.5061/dryad.gqnk98szb) and on GitHub at https://github.com/rashikaranasinghe/Data_Analysis_and_Visualization_Flameback_Pigments. The raw GBS sequencing reads have been submitted to the NCBI Sequence Read Archive (SRA) under BioProject accession PRJNA1139147.
