# Supplementary material for: Biochemical and Genomic Underpinnings of Carotenoid Colour Variation Across a Hybrid Zone Between South Asian Flameback Woodpeckers
Source: Mol Ecol. 2025 Aug 19;34(19):e70084. doi: 10.1111/mec.70084 (PMC12456116; doi:10.1111/mec.70084)
Supplement: Supplementary file 1 — Data S1: mec70084‐sup‐0001‐Supinfo01.pdf. [file MEC-34-e70084-s001.pdf]

MOLECULAR ECOLOGY

Supplemental Information for:

Biochemical and Genomic Underpinnings of Carotenoid Color Variation across a Hybrid Zone between South Asian Flameback Woodpeckers

Rashika W. Ranasinghe[1], Jocelyn Hudon[2], Sampath S. Seneviratne[3], and Darren Irwin[1]*

[1] Department of Zoology and Biodiversity Research Centre, University of British Columbia, 6270 University Blvd., Vancouver, British Columbia V6T 2K9, Canada

[2] Royal Alberta Museum, 9810 103A Avenue NW, Edmonton, Alberta T5J 0G2, Canada

[3] Avian Sciences & Conservation, Department of Zoology & Environment Sciences, Faculty of Science, University of Colombo, Colombo, Sri Lanka

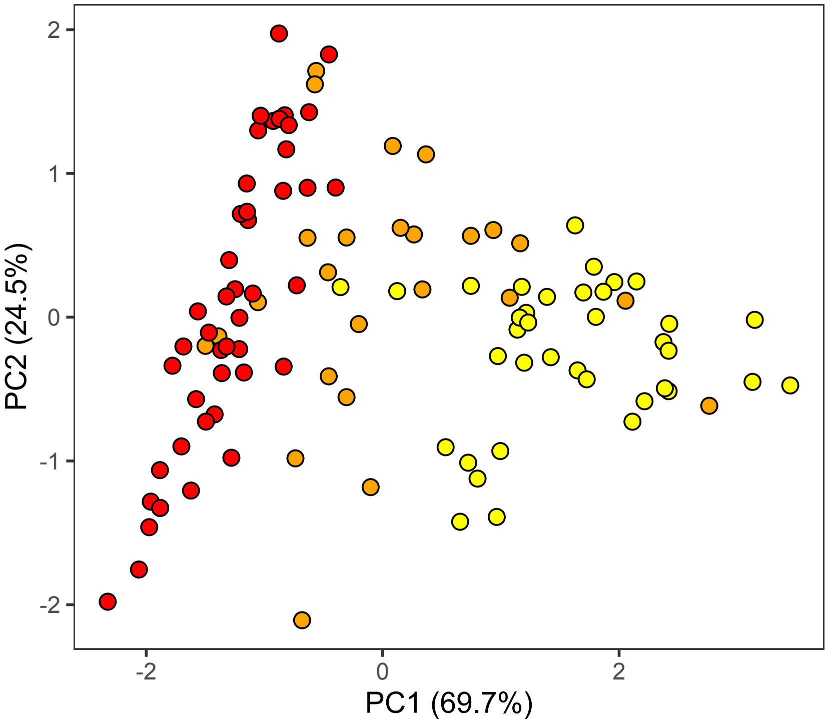

Figure S1: Principal Component Analysis (PCA) based on L, a, b colorimetric values. Principal Component (PC) 1 was utilized as the phenotypic score for the GWAS analysis. Colors denote different phenotypic groups: red for *D. psarodes*, yellow for *D. benghalense*, and orange for intermediate-plumaged flamebacks.

Classification: Protected A

MOLECULAR ECOLOGY

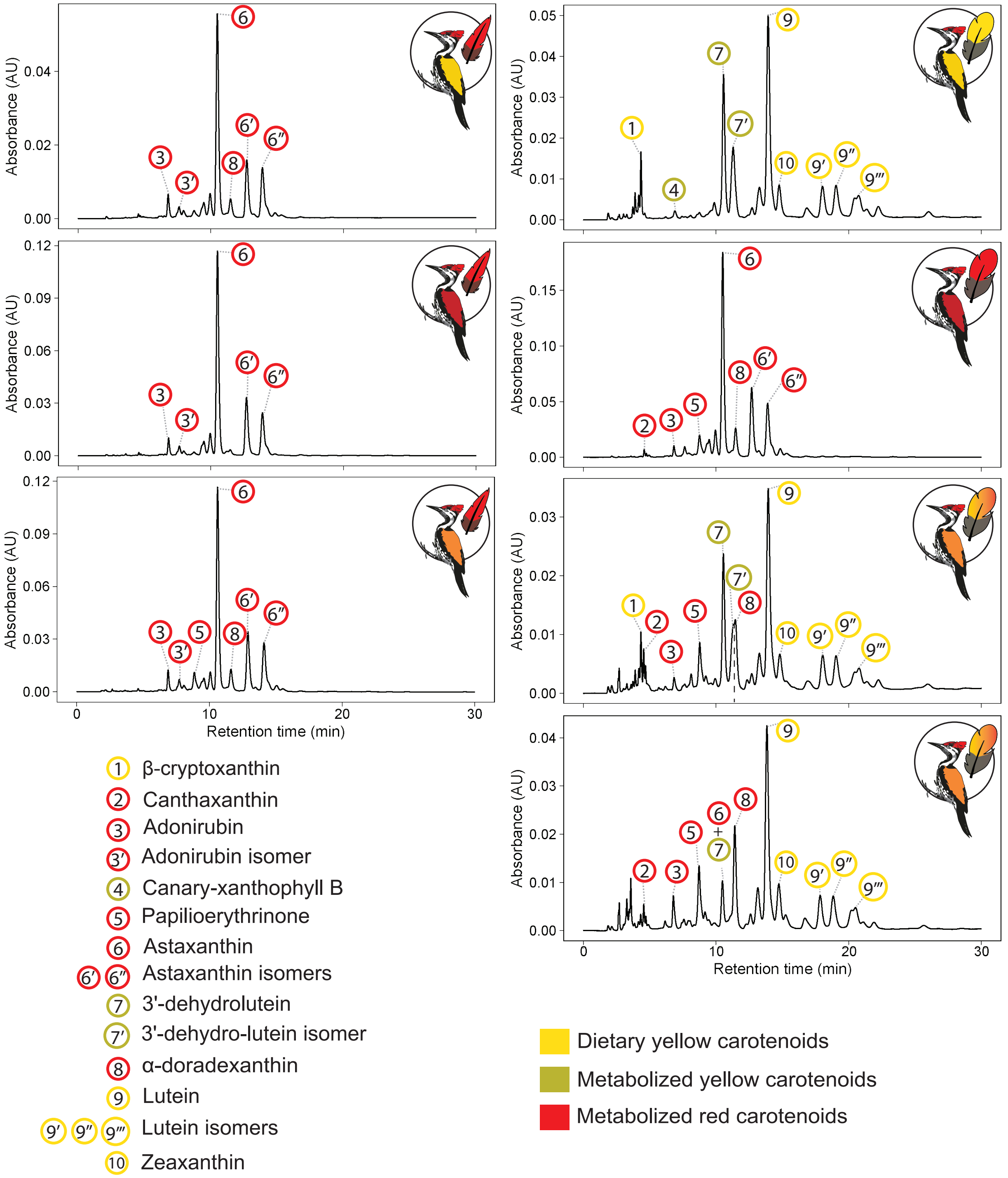

Figure S2: Examples of HPLC chromatograms at 450 nm of carotenoids in crown (left) and mantle (right) feathers from the three phenotypic groups. Colors indicate carotenoid types: light yellow for dietary yellow carotenoids, dark yellow for metabolized yellow carotenoids, and red for metabolized red carotenoids.

Classification: Protected A

MOLECULAR ECOLOGY

Sample	β-cryptoxanthin	Zeaxanthin	Lutein	3′-dehydrolutein	Canary xanthophyll A	Canary xanthophyll B	α-doradexanthin	Papilioerythrinone	Canthaxanthin	Adonirubin	Astaxanthin
UC12RR02	2.1	0	17.8	0	0	0	15.4	6.7	4.4	125.4	185.3
UC14RR01	0.9	0	5.16	0	0	0	16.9	14.5	5.1	20.9	129.8
UC14RR04	0.9	0	7.08	0	0	0	63.8	21.2	5.3	25.5	138.2
UC27RR01	1	0	6.82	0	0	0	29.1	12.6	5.3	74.1	233.5
UC28RR02	3.1	0	17.05	0	0	0	46.6	27.7	5.3	67	82
UC29RR01	0.8	0	4.13	0	0	0	30.4	14.7	5.1	26.5	159.1
UE07RR05	0.7	0	11.7	0	0	0	21.1	21.3	2.9	10.2	57.8
UF01SS02	1.2	0	3.93	0	0	0	14	9	2.5	131.2	176.3
UF01SS03	0.3	0	1.42	0	0	0	4.4	2.1	1	21	107.5
UF01SS05	1.1	0	3.74	0	0	0	22	11.2	5.6	17.3	217.7
UF02SS01	0.6	0	7.98	0	0	0	39.5	23.7	4.1	9.6	235.4
UF02SS03	0.5	0	2.7	0	0	0	15.7	4	3.7	12.2	152.1
UF02SS04	0.5	0	5.12	0	0	0	14.9	6.1	3.3	12.2	217.5
UF03RR01	0.4	0	2.02	0	0	0	4.9	2.5	1	4.9	69.5
UC20RR04-1	3	27	11.3	8.4	3.5	2.7	46.2	22.2	4.9	7.8	11.1
UC20RR04-2	21.3	23	14.72	56.9	2.5	2.6	14.5	11.4	7	3.4	0
UC20RR04-3	18.8	15.2	20.82	34.9	2.2	2.3	6.1	3.2	3.1	1.3	0
UC20RR08	13	27.9	153.3	83	2.2	1.5	39	23	10.5	3.5	0
UC29RR04	4.7	53.8	192.7	15.5	8.4	6.1	85.4	45.6	7.1	12.2	24.4
UC30RR01-1	6.2	28	205.3	62.8	5.6	6.7	51.4	60.5	4.6	6.4	0
UC30RR01-2	4	17.4	93.9	30.7	3.4	4	30.3	36.9	4.8	4.8	0
UD23RR01-1	16.5	19.1	110.3	110.6	13.4	23.3	13.5	11.3	2.6	0	0
UD23RR01-2	6	6.8	32.1	37.4	5	7.5	4.1	3.9	1	0	0
UE07RR01-1	10.1	18.3	93.1	50.7	1.7	1.2	27.7	16.2	7.1	2.6	0
UE07RR01-2	7.3	25.4	137.8	54.8	1.8	1.3	28.2	17.4	3.3	1.5	0
UE07RR03	3.2	18.9	84.2	46.6	1.4	1.5	29.3	14.3	2	1.2	0
UC19RR02	8.1	22.5	0	90.4	2.5	6.4	0	0	0	0	0
UC19RR03	5.6	6.4	10.61	39.9	1	2.2	0	0	0	0	0
UC20RR02	21.2	29.9	27.95	88.2	2.5	4.8	0	0	0	0	0
UC20RR03	12.2	16.7	12.97	55.6	1.6	2.7	0	0	0	0	0
UC20RR05	51.1	24.5	119.2	165.9	5.8	10.9	0	7.9	0	0	0
UC20RR06	8.2	6.5	26.5	24.1	2.5	3.9	0	0	1.3	0	0
UC20RR07	13.4	23	212.6	36.8	6.3	4.5	0	2.1	1.2	0	0
UC28RR01	13.7	16.7	82.3	63.7	4.7	5.8	0	9.3	2.9	0	0
UD30RR01	23.5	25.3	97.2	76.5	1.2	2.2	0	0	2.4	0	0
UD30RR03	9.2	15.3	84.7	68.9	3.5	6.7	0	0	0	0	0
UD30RR04	5.2	12.4	110.9	34.6	1	1.8	0	0	0.8	0	0
UE01RR01	20.8	16.7	124.3	45	3.7	2.3	0	0	3.2	0	0
UE01RR02	5.8	14.8	133.5	23.8	3.2	0.8	0	0	3.6	1.2	0
UE01RR03	5.5	11.6	101	40.9	2.4	3.4	0	0	0	0	0
UE01RR04	2.2	16.5	140.4	37.6	3	2.9	0	0	0	0	0
UE02RR01	12.9	20.9	182.2	31.7	5	2.6	8.5	4.7	3.6	0.7	0
UE07RR02	11.1	21.6	104.1	71.4	2.3	2.3	0	11.6	2	1	0
UE07RR04	14.3	24.3	167.3	82.2	1.9	3.2	0	7.8	1.8	1.1	0
UE09RR02	14.7	23.3	112.5	84.1	4.8	4.3	0	0	0	0	0
**Number of ε-rings**	**0**	**0**	**1**	**1**	**2**	**2**	**1**	**1**	**0**	**0**	**0**
**Number of C4-keto groups**	**0**	**0**	**0**	**0**	**0**	**0**	**1**	**1**	**2**	**2**	**2**

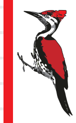

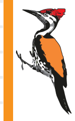

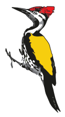

Figure S3: Absolute concentration of each carotenoid pigment (in µg/g) in each <u>mantle</u> feather of flameback woodpecker we analyzed, with the number of ε-end rings and C4-keto groups in each respective carotenoid pigment.

Classification: Protected A

MOLECULAR ECOLOGY

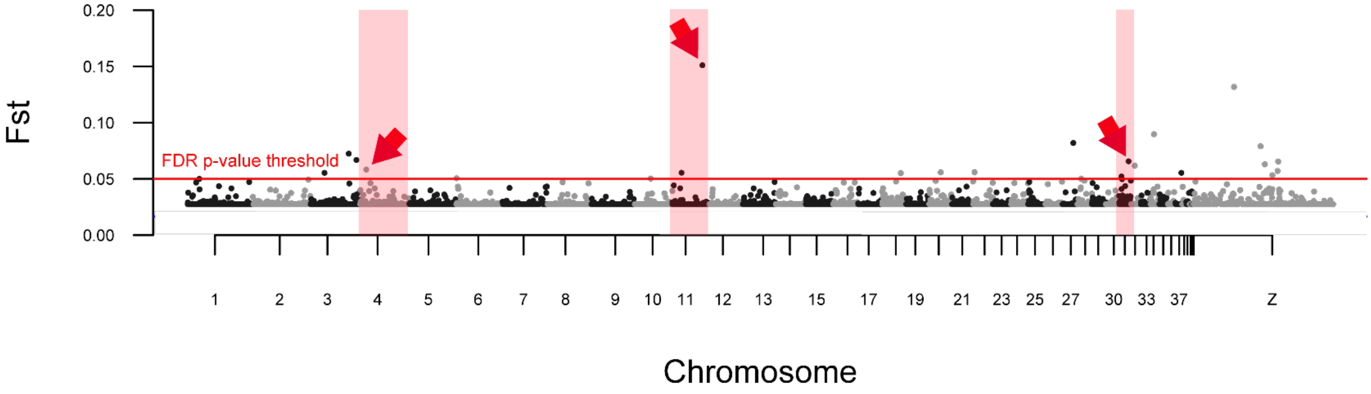

Figure S4: Genome-wide windowed F_{ST} plot. Chromosomes 4, 11 and 31 are highlighted with red bars. Red arrows indicate SNPs on these chromosomes that exceed the significance threshold (FDR adjusted p-value threshold = 0.05) for association. F_{ST} was calculated between allopatric yellow-backed *D. benghalense* and allopatric red-backed *D. psarodes* following the methodology detailed in Irwin et al. (2018). Briefly, F_{ST} values were computed across non-overlapping sliding windows of 10,000 sequenced bp throughout the genome comparing allele frequencies between populations to quantify genetic differentiation.

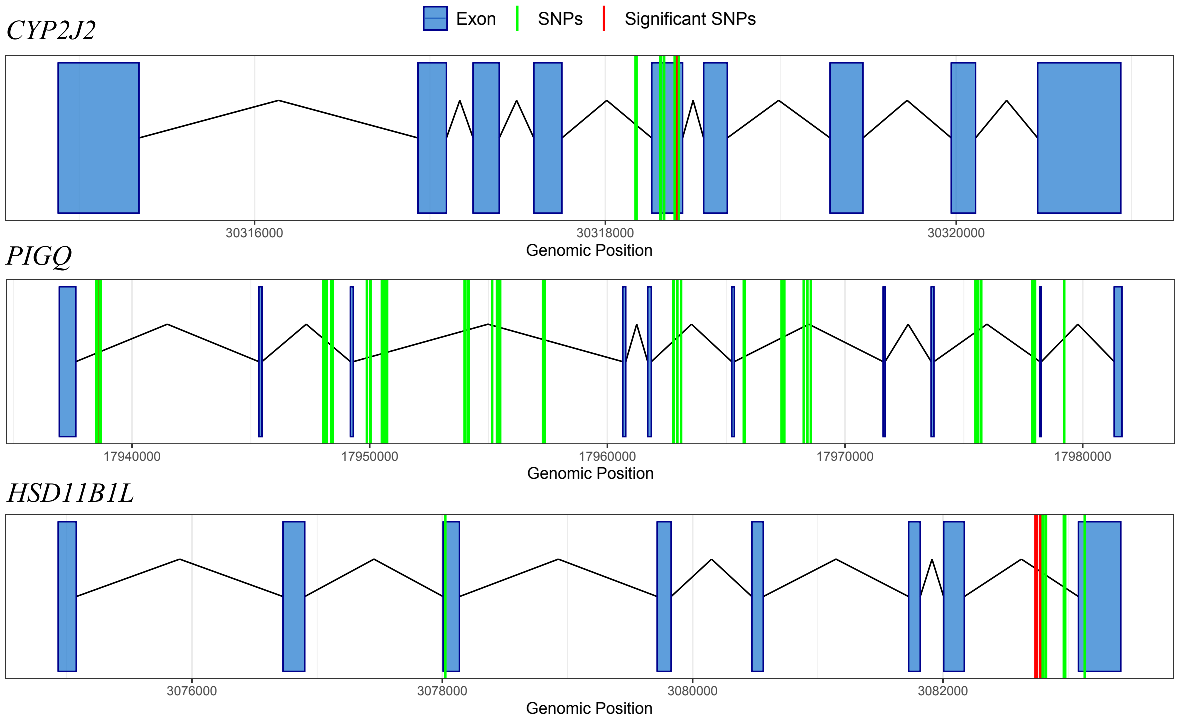

Figure S5: Gene structure diagrams for genes associated with carotenoid color expression in *Dinopium* flamebacks from the GWAS analysis. Blue boxes indicate exon regions, while red vertical lines mark the positions of significant SNPs identified in the analysis. Green lines represent additional SNPs located within the same gene.

Classification: Protected A

MOLECULAR ECOLOGY

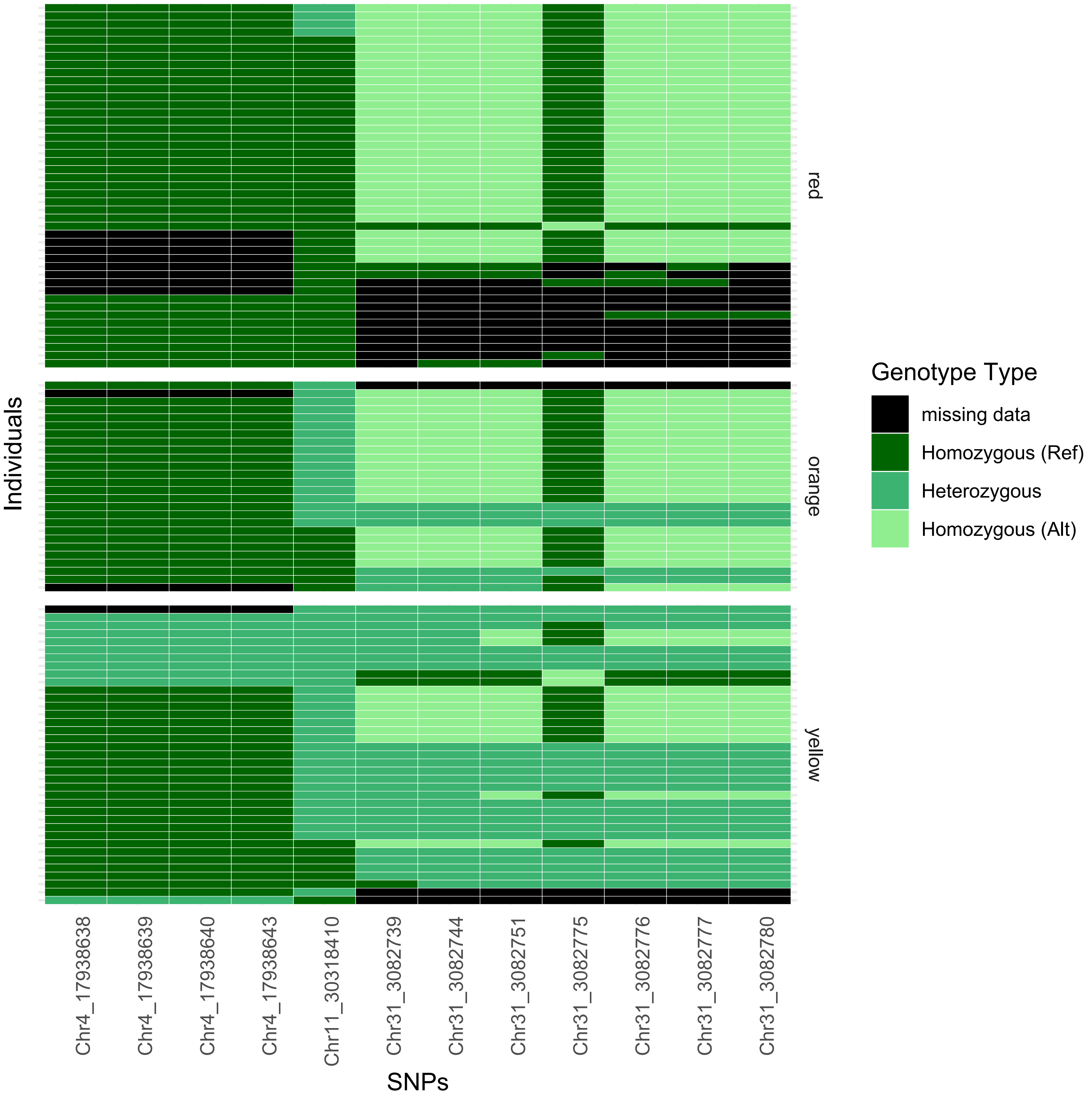

Figure S6: Genotype-by-individual plot showing the genotypes of different phenotypic groups for 12 SNPs significantly associated with carotenoid color expression in *Dinopium* flamebacks, as identified by GWAS analysis.

Classification: Protected A

MOLECULAR ECOLOGY

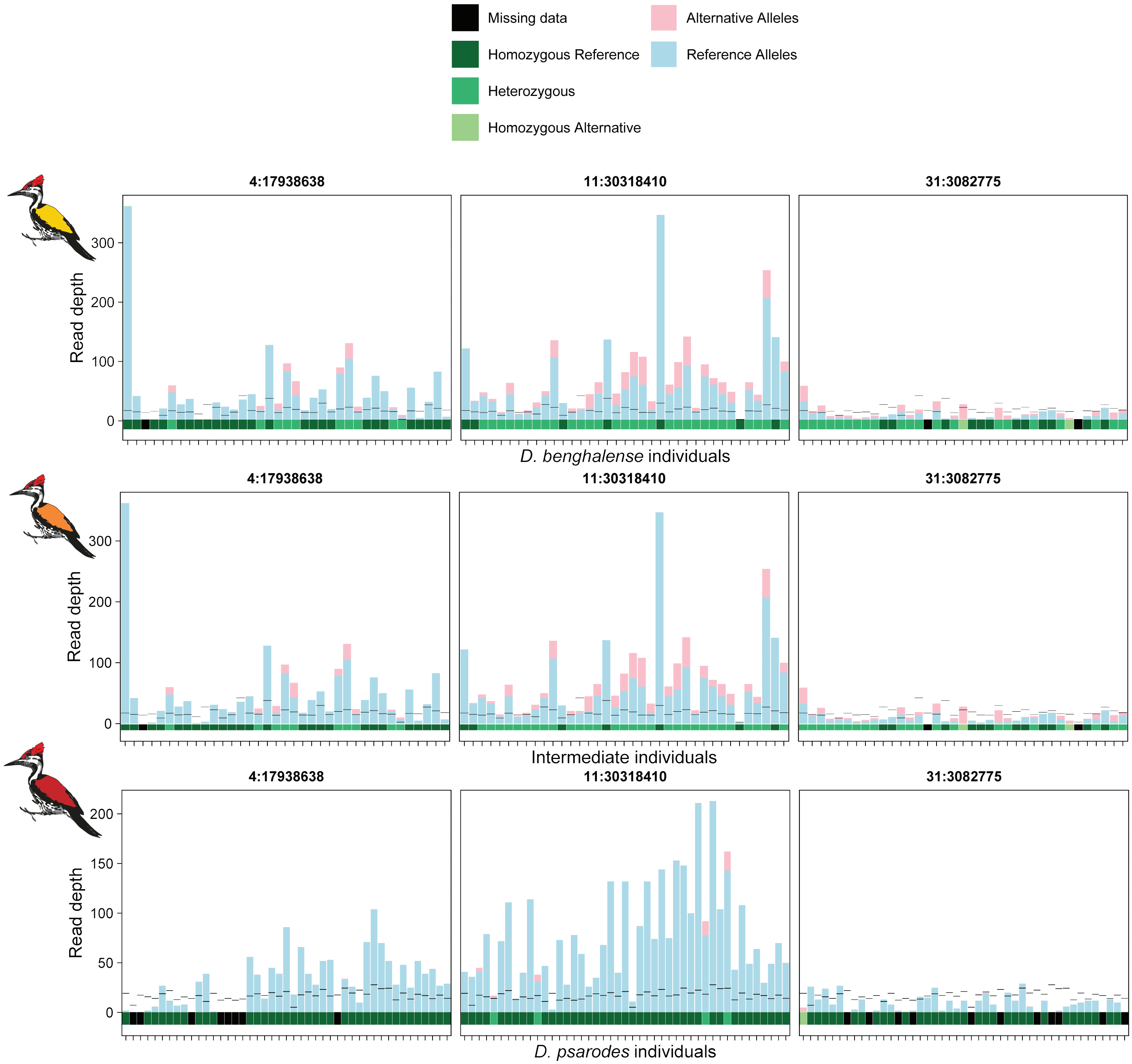

Figure S7: Read depth for each individual of SNPs on Chromosomes 4, 11 and 31. Tiles at the bottom of each plot represent the genotype for each individual at each SNP location (black for missing data, dark green for homozygous for reference allele, light green for homozygous for alternative allele and medium sea green for heterozygous individuals). Different colors on bars represent the allele type: light blue for reference allele, pink for alternative allele. The black horizontal line on each bar indicates genome-wide mean read depth of autosomes for each individual.

Classification: Protected A

MOLECULAR ECOLOGY

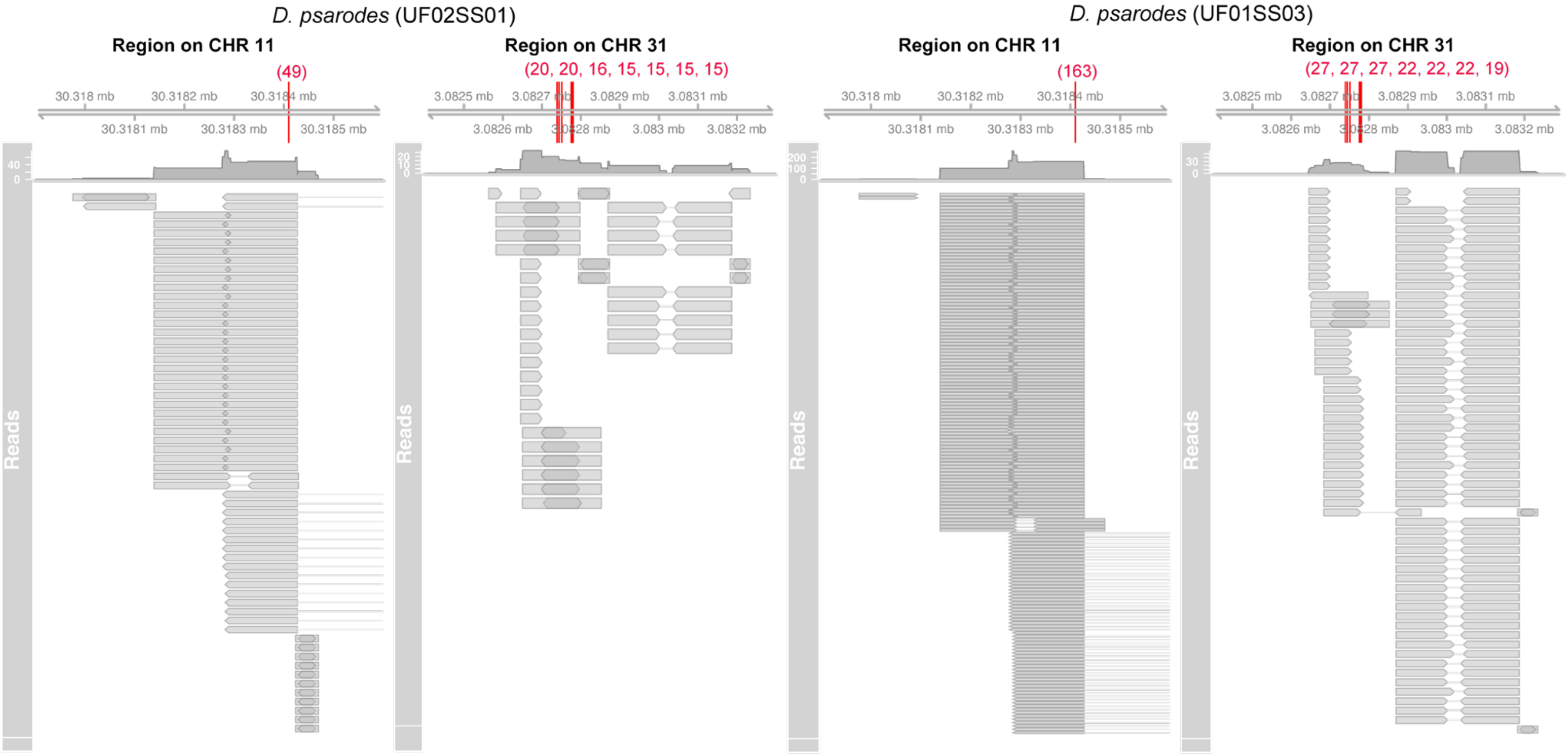

Figure S8: Raw read alignment visualization across candidate genomic regions on chromosomes 11 and 31 in two red-backed flamebacks (*D. psarodes*; individuals UF02SS01 and UF01SS03). For each individual, the left panel shows aligned reads in a region on chromosome 11 (CM026004.1: 30,317,900–30,318,600), with the focal SNP at position 30,318,410 highlighted in red. The right panel displays a region on chromosome 31 (CM026024.1: 3,082,400–3,083,300), with seven SNPs (positions 3,082,739; 3,082,744; 3,082,751; 3,082,775; 3,082,776; 3,082,777; and 3,082,780) also highlighted in red. The number of reads at each SNP is shown in red font above the highlighted positions. Histograms in the middle panel represent read depth density. Read alignments were generated using the *Gviz* package in R, based on BAM files aligned to the *Dryobates pubescens* reference genome.

Classification: Protected A

MOLECULAR ECOLOGY

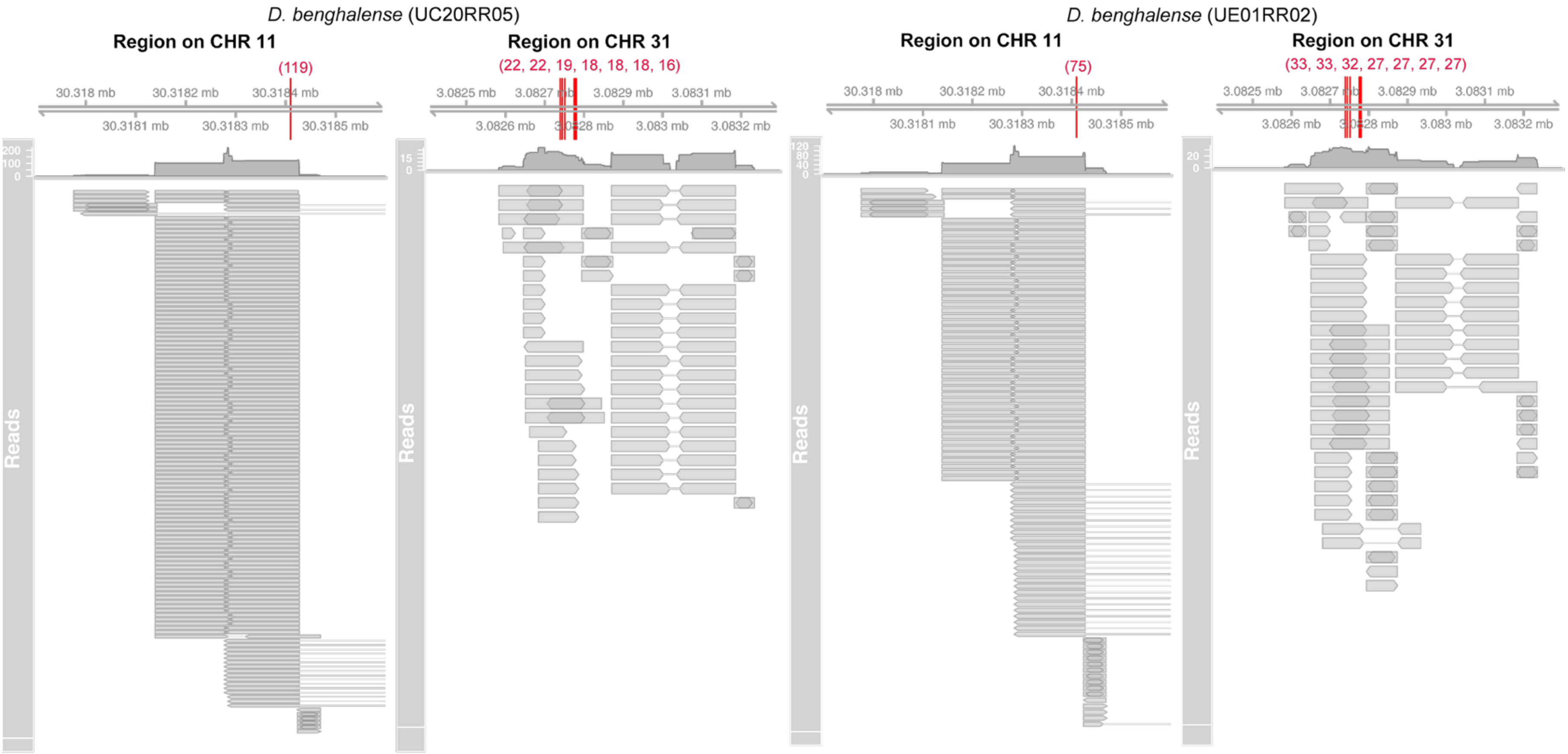

Figure S9: Raw read alignment visualization across candidate genomic regions on chromosomes 11 and 31 in two yellow-backed flamebacks (*D. benghalense*; individuals UC20RR05 and UE01RR02). For each individual, the left panel shows aligned reads in a region on chromosome 11 (CM026004.1: 30,317,900–30,318,600), with the focal SNP at position 30,318,410 highlighted in red. The right panel displays a region on chromosome 31 (CM026024.1: 3,082,400–3,083,300), with seven SNPs (positions 3,082,739; 3,082,744; 3,082,751; 3,082,775; 3,082,776; 3,082,777; and 3,082,780) also highlighted in red. The number of reads at each SNP is shown in red font above the highlighted positions. Histograms in the middle panel represent read depth density. Read alignments were generated using the *Gviz* package in R, based on BAM files aligned to the *Dryobates pubescens* reference genome.

Classification: Protected A

MOLECULAR ECOLOGY

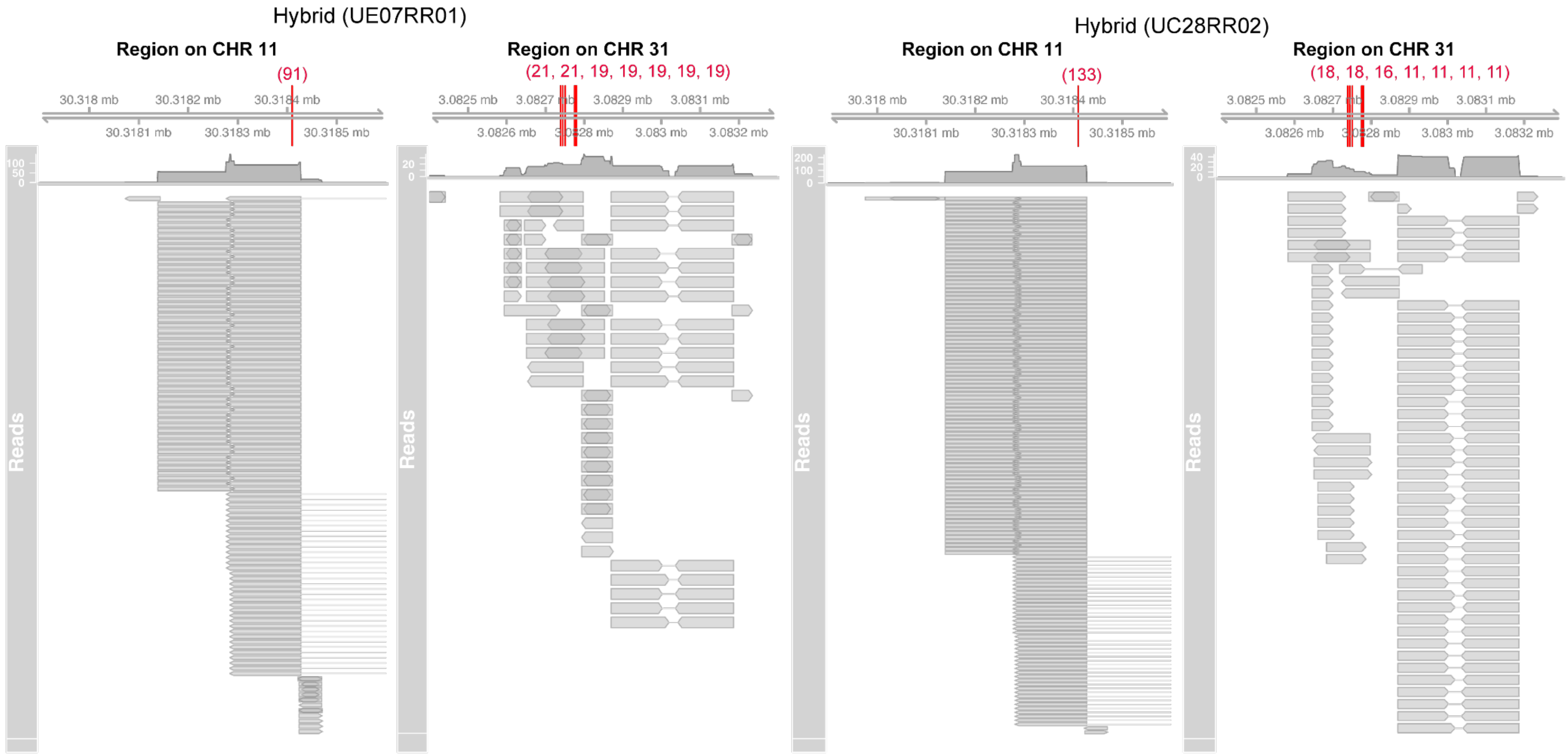

Figure S10: Raw read alignment visualization across candidate genomic regions on chromosomes 11 and 31 in two orange-backed flamebacks (Hybrids; individuals UE07RR01 and UC28RR02). For each individual, the left panel shows aligned reads in a region on chromosome 11 (CM026004.1: 30,317,900–30,318,600), with the focal SNP at position 30,318,410 highlighted in red. The right panel displays a region on chromosome 31 (CM026024.1: 3,082,400–3,083,300), with seven SNPs (positions 3,082,739; 3,082,744; 3,082,751; 3,082,775; 3,082,776; 3,082,777; and 3,082,780) also highlighted in red. The number of reads at each SNP is shown in red font above the highlighted positions. Histograms in the middle panel represent read depth density. Read alignments were generated using the *Gviz* package in R, based on BAM files aligned to the *Dryobates pubescens* reference genome.

Classification: Protected A

MOLECULAR ECOLOGY

Table S1: Results of the K-Sample Fisher-Pitman permutation test comparing carotenoid functional groups across phenotypic groups for crown and mantle feathers. P-values < 0.05 are highlighted in gray.

Feather	Functional Groups	Test statistic (Chi-squared)	p-value
Crown	C3(3')- oxygenated groups	1.197899	0.644189
Crown	ε-end rings	2.470078	0.306403
Crown	C4(4')-keto groups	2.071843	0.376286
Mantle	C3(3')-oxygenated groups	10.53059	0.002077
Mantle	ε-end rings	17.68014	0.000044
Mantle	C4(4')-keto groups	40.92194	< 0.0001

Table S2: Post-hoc analysis with pairwise tests results for functional group comparisons in mantle feathers between phenotypic groups. P-values < 0.05 are highlighted in gray.

Variable	Comparison	Test statistic	p-value	p-adjusted (FDR)
C3(3')- oxygenated groups	*D. benghalense* - Intermediates	2.6810	0.1016	0.1016
C3(3')- oxygenated groups	*D. benghalense - D. psarodes*	9.3360	0.0022	0.0067
C3(3')- oxygenated groups	Intermediates - *D. psarodes*	2.8540	0.0912	0.1016
ε-end rings	*D. benghalense* - Intermediates	0.2999	0.5839	0.5839
ε-end rings	*D. benghalense - D. psarodes*	13.1800	0.0003	0.0004
ε-end rings	Intermediates - *D. psarodes*	14.3100	0.0002	0.0004
C4(4')-keto groups	*D. benghalense* - Intermediates	10.2300	0.0014	0.0014
C4(4')-keto groups	*D. benghalense - D. psarodes*	30.7200	0.0000	0.0000
C4(4')-keto groups	Intermediates - *D. psarodes*	21.9000	0.0000	0.0000

Classification: Protected A
